# Single-cell immunophenotyping of the skin lesion erythema migrans identifies IgM memory B cells

**DOI:** 10.1172/jci.insight.148035

**Published:** 2021-06-22

**Authors:** Ruoyi Jiang, Hailong Meng, Khadir Raddassi, Ira Fleming, Kenneth B. Hoehn, Kenneth R. Dardick, Alexia A. Belperron, Ruth R. Montgomery, Alex K. Shalek, David A. Hafler, Steven H. Kleinstein, Linda K. Bockenstedt

**Affiliations:** 1 Department of Immunobiology,; 2 Department of Pathology, and; 3 Department of Neurology, Yale School of Medicine, New Haven, Connecticut, USA.; 4 Broad Institute of MIT and Harvard University, Cambridge, Massachusetts, USA.; 5 Mansfield Family Practice, Storrs, Connecticut, USA.; 6 Department of Internal Medicine, Yale School of Medicine, New Haven, Connecticut, USA.; 7 The Ragon Institute of MGH, MIT and Harvard, Cambridge, Massachusetts, USA.; 8 Institute for Medical Engineering & Science, Department of Chemistry, and Koch Institute for Integrative Cancer Research, MIT, Cambridge, Massachusetts, USA.; 9 Interdepartmental Program in Computational Biology and Bioinformatics, Yale University, New Haven, Connecticut, USA.

**Keywords:** Immunology, Infectious disease, Adaptive immunity, Bacterial infections

## Abstract

The skin lesion erythema migrans (EM) is an initial sign of the *Ixodes* tick–transmitted *Borreliella* spirochetal infection known as Lyme disease. T cells and innate immune cells have previously been shown to predominate the EM lesion and promote the reaction. Despite the established importance of B cells and antibodies in preventing infection, the role of B cells in the skin immune response to *Borreliella* is unknown. Here, we used single-cell RNA-Seq in conjunction with B cell receptor (BCR) sequencing to immunophenotype EM lesions and their associated B cells and BCR repertoires. We found that B cells were more abundant in EM in comparison with autologous uninvolved skin; many were clonally expanded and had circulating relatives. EM-associated B cells upregulated the expression of MHC class II genes and exhibited preferential IgM isotype usage. A subset also exhibited low levels of somatic hypermutation despite a gene expression profile consistent with memory B cells. Our study demonstrates that single-cell gene expression with paired BCR sequencing can be used to interrogate the sparse B cell populations in human skin and reveals that B cells in the skin infection site in early Lyme disease expressed a phenotype consistent with local antigen presentation and antibody production.

## Introduction

Lyme disease (LD), an *Ixodes* tick–transmitted infection with spirochetes of the *Borrelia burgdorferi* *sensu lato* complex (genus *Borreliella*; ref. 1), is now the most common vector-borne disease in the Northern Hemisphere (2, 3). The earliest sign of infection reported in approximately 70% of patients is the appearance of an erythema migrans (EM) skin lesion at the initial tick bite site (3). EM presents as an expanding, erythematous macule that usually appears 1–2 weeks after infection, and arises from the immune response to *B*. *burgdorferi* spirochetes as they disseminate outwardly from the tick bite site (3). If not controlled by the local immune response in the skin, *B*. *burgdorferi* disseminates via the lymphatics and blood to cause disease in other areas of the skin and internal organs, including the heart, musculoskeletal, and nervous systems (3).

*Borreliella* spirochetes are widely held to be extracellular pathogens against which phagocytes, antibody, and complement are critical for host defense (4). *B*. *burgdorferi*–specific antibodies prevent infection in both animal models and humans and contribute to a reduction in pathogen burden and resolution of disease manifestations in mouse models (4). In humans, a robust circulating plasmablast response was found to correlate with more rapid resolution of LD symptoms after antibiotic treatment for EM (5). Within the EM lesion itself, however, B cells and plasma cells are not a dominant cell type. Histologic studies of EM reveal superficial and deep perivascular mononuclear infiltrates of lymphocytes and macrophages, occasional plasma cells, and, less commonly, neutrophils and mast cells (6, 7). By IHC, CD3^+^ T cells predominate the immune infiltrate in comparison with CD68^+^ macrophages or CD20^+^ B cells (6). The prevalence of B cells in EM lesions, however, may relate to the duration of the lesion prior to sampling. In EM lesions arising from infection with *Borreliella* strains in Europe, the proportion of infiltrate due to B cells was found to increase with the density of the infiltrate and size of the EM lesion, which typically reflects lesion duration (8). A study using a blister technique to extract cells from EM lesions found an increased frequency of T cells in comparison with their prevalence among PBMCs (9). In contrast, B cells were found at a frequency that was considerably lower than their prevalence in PBMCs, although this could be influenced by the method for cell extraction. By flow cytometry, both CD4^+^ and CD8^+^ T cells appeared antigen experienced, as assessed by CD45RO and CD27 expression, with enrichment of CD4^+^ T memory and effector subsets and CD8^+^ effector/cytolytic subsets. Blister fluid also contained elevated levels of proinflammatory cytokines IL-6, TNF-α, and IFN-γ as well as the anti-inflammatory cytokine IL-10, consistent with an earlier report of elevated mRNA expression of TNF-α, IFN-γ, and IL-10 detected by in situ hybridization using cytokine-specific RNA probes (6). More recently, evaluation of the whole skin transcriptome of EM lesions identified a strong IFN signature, consistent with cell-mediated immunity coordinated by T cells and innate immune populations as the main feature of the local immune response (10). The specific contribution of B cells to the cutaneous response, however, remains an open question. Understanding B cell responses in the EM lesion is key to identifying host factors that may be important determinants of localized versus systemic infection with this extracellular pathogen.

Here, we leveraged new advances in single-cell RNA-Seq (scRNA-Seq) analysis to characterize the B cell response in the EM lesion. Using the 10× Genomics Chromium scRNA-Seq platform, we defined the transcriptomes of single cells from whole-skin digests of EM and autologous uninvolved skin biopsies from patients with LD. Single-cell transcriptomics was combined with B cell receptor (BCR) and T cell receptor (TCR) repertoire sequencing to determine the origin and trafficking of these immune populations between the circulation and the skin. We found that clonally expanded B cells bearing plasma cell as well as memory B cell signatures were recruited to the site of the EM lesion, accompanied by activated T cells and myeloid subsets. Compared with B cells from uninvolved skin, EM B cells upregulated MHC class II genes and displayed a biased VH-gene usage compared with circulating B cells, suggesting an antigen-specific response. A disproportionate fraction of memory B cells utilized IgM receptors and displayed a low frequency of somatic hypermutation (SHM). This IgM-expressing B cell subset did not appear to be naive, but rather it was characterized by a TLR activation signature. Notably, clonal relatives of these B cells were found in the circulation. Taken together, our findings support a role for B cells, including IgM-expressing memory B cells, in local antigen presentation and antibody production in the skin response to *B*. *burgdorferi* infection.

## Results

### Subject characteristics and sample collection.

To identify and immunophenotype cell subsets in EM lesions at a single-cell level, we obtained biopsies of EM lesions from 10 subjects with early LD separated into 2 cohorts based on year of enrollment (Table 1). All subjects satisfied the CDC LD case definition; 9 of the 10 tested positive for *B*. *burgdorferi* antibodies using the C6 peptide enzyme immunoassay (Table 1; refs. 11–13). One subject in cohort 1 had disseminated infection manifesting as multiple EM without clinically apparent disease in extracutaneous sites, whereas the remainder had a single EM. Biopsies of uninvolved skin were obtained from 5 of 6 subjects in cohort 1 and from 2 of 4 subjects in cohort 2 (Table 1).

### Skin cell cluster identification and preliminary characterization using single-cell transcriptomics.

**Figure 1:**
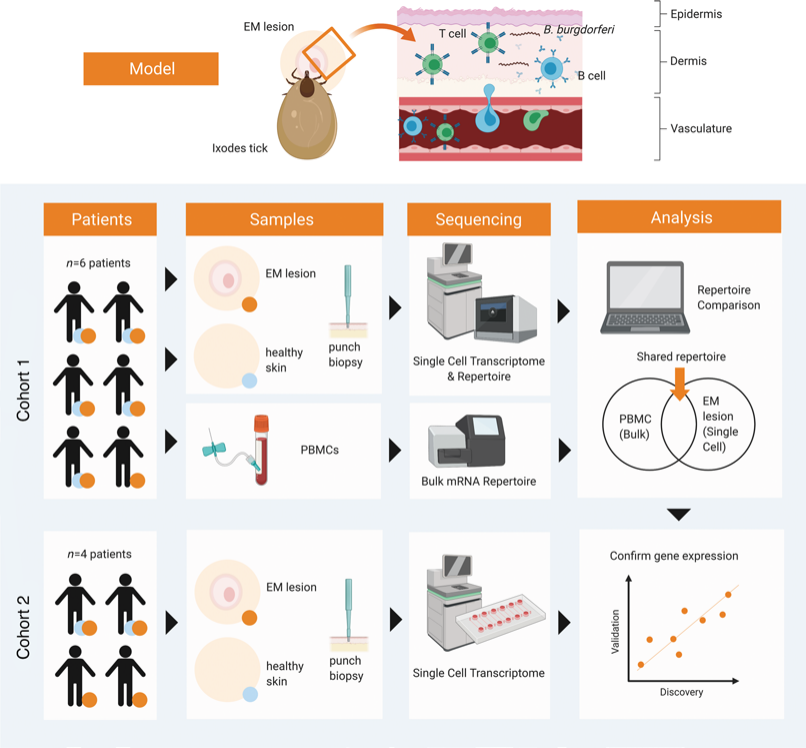
Schematic demonstration of study workflow. EM and uninvolved skin samples were first isolated from patients by punch biopsy and submitted for scRNA-Seq. In cohort 1 (*n* = 6), paired PBMCs were prepared as unpaired bulk TCRB and IGH repertoires from bulk RNA and also submitted for repertoire sequencing using the 10× Genomics Chromium platform. The characteristics of B cells and T cells found in both compartments were analyzed based on gene expression and features of their respective V(D)J sequences. Cohort 2 (*n* = 4) subject samples were sequenced using a nanowell-based single-cell transcriptome approach (Seq-Well) to support findings from cohort 1. EM, erythema migrans; sc, single-cell.

**Figure 2:**
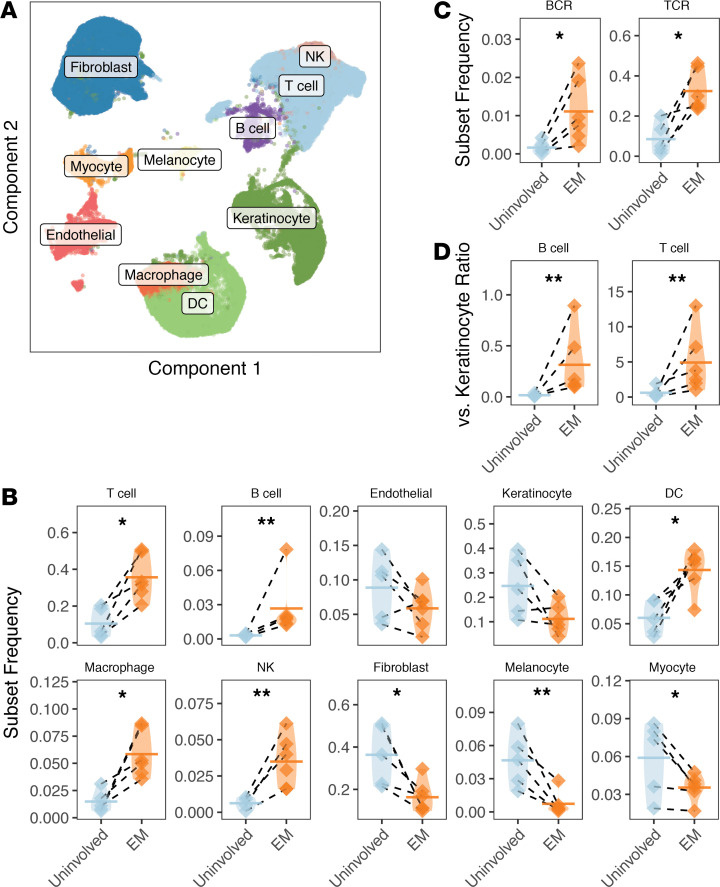
B cells and T cells infiltrate EM lesions. (**A**) UMAP projection of single-cell gene expression data from both EM and uninvolved samples from subjects in cohort 1 with clusters annotated based on marker genes. (**B**) The fraction of cells found in each cluster per subject as a fraction of total cells from the sample for all 10 cell subsets (as annotated by marker genes). (**C**) Frequency of cells associated with a reconstructed IGH and associated IGK/L (“BCR”) or TCRB (“TCR”) receptor from repertoire sequencing as a fraction of total cells in the sample. (**D**) Ratio of B cells and T cells relative to keratinocytes in EM lesions compared with uninvolved samples based on frequencies computed from data in **B**. Horizontal bars show the mean frequency of each comparison and frequencies belonging to the same subject are connected with dashed lines. Data for the same *n* = 6 subjects from cohort 1 are shown for all panels. Statistical differences are shown only when significant for a paired ratio *t* test (**P < 0.01; *P < 0.05). EM, erythema migrans; BCR, B cell receptor; TCR, T cell receptor.

Single-cell suspensions of fresh EM and uninvolved skin samples from subjects in cohort 1 were analyzed by 10× Genomics scRNA-Seq with paired transcriptomes and repertoires without selecting for specific cell populations (Figure 1 and Supplemental Table 1; supplemental material available online with this article; https://doi.org/10.1172/jci.insight.148035DS1). Single-cell transcriptomes (Supplemental Figure 1, A–C) were pooled across subjects to identify cell clusters, to which identities were assigned (Supplemental Figure 2A) based on the expression of key marker genes for stromal and immune cells (Supplemental Figure 3 and Supplemental Table 2). As expected, there was increased representation of immune cell subsets, especially lymphocytes, in the EM skin versus adjacent uninvolved skin (Figure 2, A and B). On average, T cells constituted 35.6% of EM cells versus 10.5% of uninvolved skin cells (paired ratio *t* test, *P* = 0.012). B cells, which sparsely populate normal skin (14), accounted for 2.66% of EM lesion cells in comparison with 0.31% of cells from uninvolved skin samples (Figure 2B). Myeloid cells (paired ratio *t* test, *P* = 0.019), dendritic cells (paired ratio *t* test, *P* = 0.020), and NK cells (paired ratio *t* test, *P* = 0.006) were also found to be enriched in the EM lesion in comparison with uninvolved skin. The infiltration of immune cells consequently decreased proportions of nonimmune cell subsets. Fibroblasts constituted 16.3% of cells in the EM lesion versus 36.3% of uninvolved skin and keratinocytes constituted 11.2% of EM cells versus 24.6% of uninvolved skin cells (Figure 2, A and B).

Because the estimation of cell frequencies is dependent on the accuracy of cell cluster identification, we used a second method, the Seq-Well nanowell-based platform, to perform scRNA-Seq of EM and uninvolved skin samples from subjects in cohort 2 (Supplemental Table 3; ref. 15). After cluster identification, similar analyses were performed quantifying the frequency of different subsets and gene expression profiles (Supplemental Figure 4, A and B). The frequency of B cells was increased (Supplemental Figure 4, C and D) in 1 of 2 subjects that had paired samples (4.98% vs. 0.62% in EM vs. uninvolved skin, respectively), whereas no significant elevation was observed in the second. On average, EM biopsies from all 4 subjects in cohort 2 contained 2.0% B cells, similar to the 2.7% observed in cohort 1.

To more precisely define the B cell and T cell clusters, we examined their association with BCR and TCR expression by BCR and TCR repertoire sequencing using cohort 1 samples (Figure 2, B and C). These analyses could only be performed on cohort 1 samples because Seq-Well does not yet permit BCR and TCR sequencing. Both B cells (paired ratio *t* test, *P* = 0.021, 1.47% in EM, 0.16% in uninvolved) and T cells (paired ratio *t* test, *P* = 0.023, 32.5% in EM, 8.5% in uninvolved) identified using this new definition were statistically more abundant in the EM lesion in comparison with uninvolved skin (Figure 2C). Differences between the frequency of B cells and T cells in uninvolved compared with EM skin also were significant when calculated as a ratio relative to the frequency of keratinocytes, irrespective of whether the definition of B cells and T cells was based on marker genes (Figure 2D) or repertoire sequencing (Supplemental Figure 5). Taken together, our results show that B cells were in greater abundance in EM compared with uninvolved skin, along with T cells and innate immune cell subsets. T cells were the dominant lymphocyte subset, consistent with observations from earlier studies (8, 9).

### B cells in EM lesions upregulated MHC class II expression.

**Figure 3:**
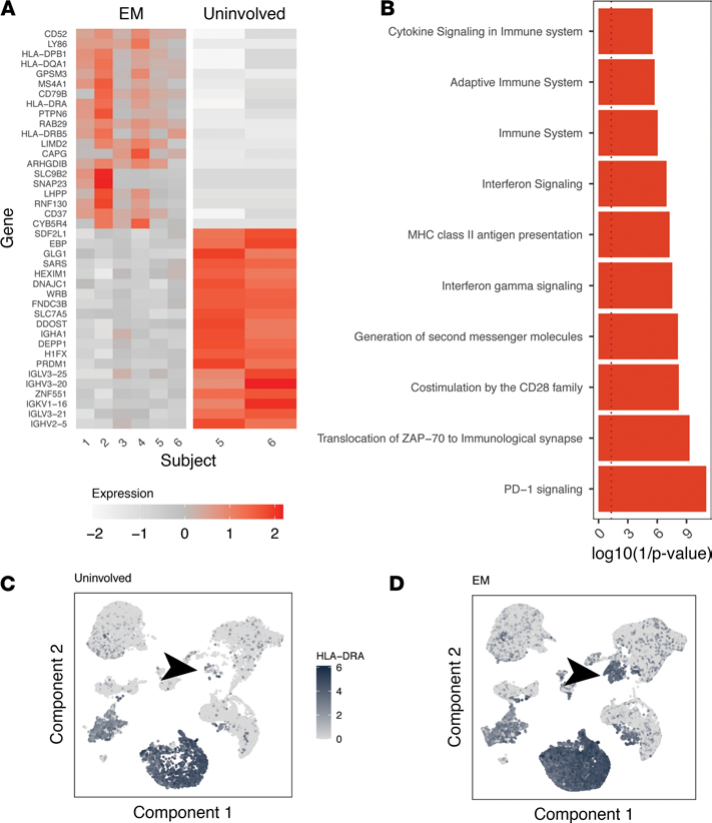
B cells in EM lesions express high levels of MHC class II genes and signatures of IFN gene signaling and antigen presentation. (**A**) Heatmap of top 20 and bottom 20 DEGs for B cells from EM compared with B cells from uninvolved skin based on absolute average minimum gene expression difference (see Methods). (**B**) enrichR gene ontology analysis of the top 40 genes upregulated in B cells from EM tissue. Red bars correspond to the top significantly associated gene ontology assignments (*P* < 0.05 by Wilcoxon’s test). Gene expression of the “HLA-DRA” MHCII gene displayed over a UMAP projection of all single-cell gene expression data from the 6 subjects with the intensity of shading correlated with the normalized expression in either uninvolved (**C**) or EM skin (**D**). The arrow points to the location of the B cell single-gene expression cluster. Data for the same *n* = 6 subjects from cohort 1 are shown for all panels. EM, erythema migrans; DEGs, differentially expressed genes.

To begin to phenotype B cells within the EM lesions, we investigated the genes most differentially expressed between B cells from 6 EM samples and 2 uninvolved skin samples by absolute difference using pseudo-bulk gene expression profiles. We were unable to perform paired testing for significance because no B cells with associated IGH were identified in the other 3 uninvolved skin samples. In the samples in which B cells were detected, 9 MHC class II genes were expressed by B cells, 4 of which were in the set of the top 20 upregulated genes (Figure 3A). Overrepresentation analysis using the top 40 differentially expressed genes (DEGs) and the Reactome pathways database identified a significant signature for MHC class II antigen presentation (*P* = 1.2 × 10^–5^), along with a signature for IFN signaling (*P* = 2.1 × 10^–5^) and IFN-γ signaling (*P* = 7.4 × 10^–6^; Figure 3B and https://doi.org/10.5281/zenodo.4711465). Visualization of the expression of the MHC class II gene HLA-DRA confirmed its low expression in the B cell cluster in uninvolved skin (Figure 3C) compared with its prominent upregulation in EM (Figure 3D).

### T cells displayed a signature of trafficking and receiving costimulation.

We also examined the differences between T cell subsets in EM compared with uninvolved skin. Due to T cell heterogeneity, we first assigned T cells with an associated TCRB V(D)J sequence to either a CD4^+^ or CD8^+^ cell cluster computationally (Supplemental Table 4), followed by Tregs and dividing T cell subsets using a set of lineage-defining marker genes (Supplemental Figure 6, A–C, and Supplemental Table 5; ref. 16). The presence of CD4^+^ T cells in the 5 EM samples that had paired uninvolved skin samples permitted paired significance testing of pseudo-bulk gene expression profiles. Using a FDR threshold of 0.1, a total of 2944 genes were differentially expressed by CD4^+^ T cells in EM lesions compared with matched uninvolved skin (Supplemental Figure 7A). Signatures enriched among upregulated genes in EM skin included costimulation by the CD28 family (*P* = 0.006) and chemokine receptors bind chemokines, consistent with interactions between CD4^+^ T cells and antigen-presenting cells (APCs) in the skin microenvironment (Supplemental Figure 7D and https://doi.org/10.5281/zenodo.4711465). No significantly DEGs were identified for CD8^+^ T cells (https://doi.org/10.5281/zenodo.4711465; 4 sets of paired samples) or Tregs (https://doi.org/10.5281/zenodo.4711465; 4 sets of paired samples) in EM versus uninvolved skin after FDR correction (Supplemental Figure 7, B and C; top 20 DEGs shown based on absolute expression differences). However, when considering the top 40 EM-associated genes, several inflammation-related gene sets were enriched in CD8^+^ T cells and Tregs (Supplemental Figure 8, A and B).

The pattern of gene expression among the most highly changed genes in the EM skin versus uninvolved skin was similar between cohort 1 and cohort 2. We found a significant correlation when comparing the absolute expression differences of the top 20 upregulated and downregulated genes in cohorts 1 and 2 for CD4^+^ T cells (Supplemental Figure 9A) as well as CD8^+^ T cells (Supplemental Figure 9B), Tregs (Supplemental Figure 9C), and B cells (Supplemental Figure 9D). The same positive correlation was also observed in myeloid cells (Supplemental Figure 9E and https://doi.org/10.5281/zenodo.4711465). In cohort 2, we again observed B cell upregulation of the MHC class II gene HLA-DQA2 and CD69, a marker of both cell activation and resident tissue cells (17). The findings that EM B cells increased the expression of genes involved in antigen presentation and interaction with T cells, particularly MHC class II genes, and EM T cells upregulated genes associated with costimulation and interactions with APCs support a role for B cells as APCs in the EM lesion.

### Memory B cells, including unmutated class-switched B cells, were enriched in EM lesions.

**Figure 4:**
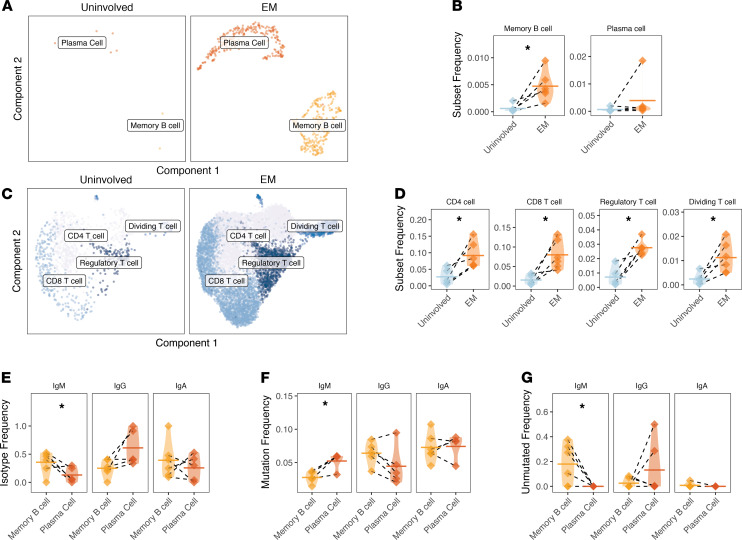
Memory B cells and all T cell subsets are more abundant in EM skin; memory B cells have distinct characteristics compared with plasma cells. (**A**) UMAP projection of B cell subsets from uninvolved or EM skin. Cells include those assigned to the B cell cluster with an associated BCR from repertoire sequencing. (**B**) Frequency of cells from the 2 B cell clusters reported as a frequency of total cells, with significance shown for a ratio *t* test. One subject had a high plasma cell frequency (1.9%) largely due to the presence of an expanded clone; (**C**) UMAP projection of T cell subsets from either uninvolved or EM skin. Cells include those assigned to the T cell cluster with an associated TCRB receptor from repertoire sequencing. (**D**) Frequency of cells from the 4 T cell clusters as a frequency of total cells from each subject sample from either uninvolved or EM skin with significance shown as a ratio *t* test. (**E**) Average distribution of isotypes among members of the 2 EM B cell clusters, expressed as a fraction of total cells from the subset. (**F**) Average SHM frequency of 2 EM B cell clusters based on isotype. (**G**) Overall frequency of unmutated VH gene segments among members of the 2 B cell cluster by isotype, expressed as a fraction of the subset with a given isotype. Horizontal bars show the mean frequency of each comparison and frequencies belonging to the same subject are connected with dashed lines. Data for the same *n* = 6 subjects from cohort 1 are shown for all panels. Statistical differences are shown only when significant for a paired (ratio) *t* test (*P < 0.05). EM, erythema migrans; BCR, B cell receptor.

B cells in the skin belonged to 1 of 2 clusters, either a memory cell cluster or a plasma cell cluster, as identified by automated cell annotation (Supplemental Table 6) and based on the marker genes MS4A1 (CD20) for memory B cells and PRDM1 (BLIMP-1) for plasma cells (Supplemental Table 7 and Supplemental Figure 10, A and B). The frequencies of memory B cells and plasma cells (Figure 4, A and B), as well as CD4^+^ T cells, CD8^+^ T cells, Tregs, and dividing T cells (Figure 4, C and D), were quantified as a total fraction of the cells present in the biopsy sample. An enrichment for memory B cells (paired ratio *t* test, *P* = 0.010; Figure 4B) and all T cell subsets (paired ratio *t* test for CD4^+^ T cells, CD8^+^ T cells, Tregs, and dividing T cells were *P* = 0.019, *P* = 0.017, *P* = 0.014, *P* = 0.017, respectively; Figure 4D) was observed. Although the mean frequency of plasma cells was also higher in EM skin, this was not statistically significant (paired ratio *t* test, *P* = 0.126; Figure 4B) and appeared to be driven by a single subject due to the presence of 1 expanded clone; requantification excluding this subject confirmed no significant influx of plasma cells into the lesion (paired ratio *t* test, *P* = 0.261).

To better define the differences and respective functions between these 2 B cell subsets present in EM, we compared their BCR repertoire properties. We observed that memory B cells were more likely to be IgM (paired *t* test, *P* = 0.031; Figure 4E), and these IgM memory B cells displayed a lower frequency of SHM compared with IgM plasma cells (paired *t* test, *P* = 0.038; Figure 4F). Moreover, a subpopulation of memory B cells were unmutated (defined as <1% mutation frequency in V and J segments), particularly among IgM-expressing B cells (consistently more than plasma cells, paired *t* test, *P* = 0.046; Figure 4G and Supplemental Table 8). A subpopulation of IgG-switched plasma cells were also unmutated (5 cells across all samples or 15.8% of plasma cells; Supplemental Table 9). IgA-switched and IgG-switched cells were detected in both memory B and plasma cell populations. IgA-switched memory B cells were present in EM lesions in all subjects and among plasma cells in 4 subjects; IgG-switched cells were detected among all plasma cells in EM lesions and were only absent from the memory B cells of 1 subject. Overall, the memory B cell infiltrate in EM lesions contained a higher frequency of IgM-expressing cells than the plasma cell infiltrate.

### Evidence for antigen-driven selection among B cells and T cells.

Given that we found B cells expressing IgA- and IgG-switched BCRs in the EM infiltrate, which suggests prior antigen experience, we examined the VH gene usage in the BCR repertoire of EM lesions. VH gene usage reflects the underlying antigen specificity of B cells and differences would suggest active selection of B cells found in the tissue based on specificity. We compared the VH gene usage distribution of B cells in the EM lesion with the repertoire observed from bulk sequencing of PBMCs (Supplemental Table 10) in these same subjects. We reasoned that PBMCs would be a suitable reference because the VH gene usage of the circulating B cell repertoire has been well characterized and the blood is the most proximal location from which nonresident B cells might enter the skin.

Switched B cells (IgA or IgG) in the EM lesion expressed less IGHV4-34 (paired *t* test, *P* = 0.002; Supplemental Figure 11A) and less IGHV3 overall (paired *t* test, *P* = 0.004; Supplemental Figure 11B). However, with the exception of IGHJ1 (paired *t* test, *P* = 0.001, usage of this J gene occurred in less than 2% of the circulating repertoire), no differences in the use of IGHJ genes were observed (Supplemental Figure 11C). To confirm that the VH gene usage differences were not due to the different technologies used to profile the skin and PBMCs (single cell vs. bulk, respectively), we verified our findings using a parallel single-cell PBMC sequencing sample from 1 subject with EM (Supplemental Figure 11, D–F). These analyses confirmed that B cells in the EM lesion were clonally expanded with a distinct VH gene repertoire featuring decreased use of IGHV3 family genes.

We found additional evidence for antigen-driven selection of B cells and T cells within the EM lesion by evaluating their clonal expansion (Supplemental Figure 12, A–C). In the case of B cells, clones were expanded in EM skin compared with paired uninvolved skin. Because traditional diversity metrics could not be used due to the sparsity of B cells in uninvolved skin samples, we quantified clonality by calculating the mean size of B cell clones in each sample. The mean number of cells of the largest B cell clone in uninvolved tissue was 1 B cell (i.e., no expansions in samples where B cells were found), whereas in EM tissue, the mean size of the largest B cell clone was 39 B cells (1-tailed paired Wilcoxon’s test *P* = 0.029; Supplemental Table 11). T cells were also more clonally expanded in the EM skin. We found that the mean size of the largest CD8^+^ T cell clone was larger in EM compared with uninvolved skin (1-tailed paired Wilcoxon’s test, 20 vs. 6 cells, *P* = 0.063; Supplemental Table 12), with a similar trend also observed for CD4^+^ T cell clones (1-tailed paired Wilcoxon’s test, 23 vs. 9, *P* = 0.094; Supplemental Table 13). EM B cells were also expanded compared with a control blood B cell single-cell repertoire available from 1 subject (4.9% for the largest clone in skin and 1.4% for the largest clone in blood, consistent with sizes reported in the literature) using methods that correct for different sampling depths (18). The presence of these clonal expansions is consistent with antigen-driven selection in situ.

### EM-associated B cells and T cells were traced to the circulation.

To assess whether B cells and T cells present in EM skin were derived from a localized response in situ or active trafficking from the circulation, we examined the bulk repertoire of PBMCs (Supplemental Table 10) for clonal relatives of these EM cells in each subject. By quantifying the extent to which members of the same clonal family were found in both EM skin and blood, we found the overlap was highly significant for both B cells (paired *t* test, *P* = 0.014; Supplemental Figure 13A) and T cells (*P* = 6.1 × 10^–5^; Supplemental Figure 13B), suggesting a meaningful clonal connection between the 2 sites. EM B cells that were clonally related to circulating B cells (referred to as “mobile”) had similar repertoire properties to those without such relatives (referred to as “resident”). Specifically, no significant differences between mobile and resident B cells were found for isotype usage or SHM frequency (Supplemental Figure 14, A and B, respectively). We noted that up to 20% of the resident IgG B cells were unmutated, whereas no unmutated IgGs were found among mobile B cells, but this difference was not significant (Supplemental Figure 14C).

Because the PBMC repertoire was profiled using bulk repertoire sequencing, clones were defined for B cells and T cells using only IGH or TCRB, respectively, in these analyses. Although we have previously shown that IGH alone is sufficient to determine most B cell clonal relationships, up to 20% of clones defined this way may be subdivided based on light chain expression (19). To confirm the results obtained with bulk receptor sequencing, we examined 1 subject from whom both skin and blood B cell repertoires were obtained with paired heavy and light chain receptors. We observed that 76.5% of the clones shared between EM and the circulation and detected by examination of the heavy chain alone were preserved when the light chain was also incorporated in the clonal definition. Similar results were obtained for TCRB (85.6%). This single-cell analysis confirmed the presence of mobile B cells at the EM lesion, and that the mobile and resident B cells exhibited similar repertoire properties (Supplemental Figure 14, D–F).

**Figure 5:**
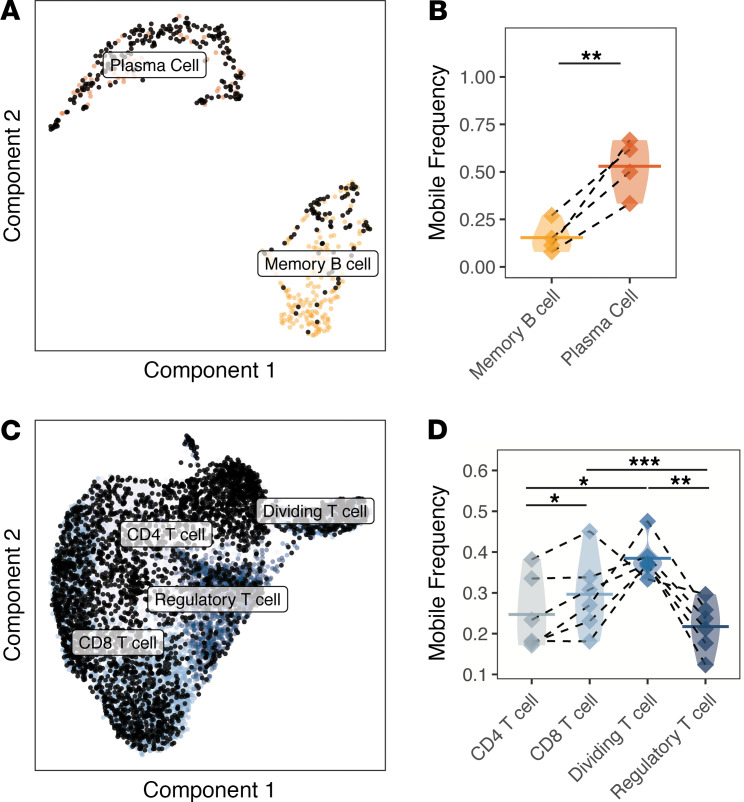
B cell and T cell clones from the EM lesions can be traced to the circulation. (**A**) UMAP projection of the B cell subset overlaid with black dots denoting B cells clonally related to those present in the circulation identified by bulk IGH repertoire sequencing (unpaired). (**B**) The frequency of each B cell subset clonally related to those present in the circulation is designated as “mobile.” Samples with fewer than 6 B cells (paired with BCR receptors) were excluded from analysis. (**C**) UMAP projection of the T cell subset overlaid with black dots denoting T cells that are clonally related to those present in the circulation from bulk TCRB repertoire sequencing (unpaired). (**D**) Frequency of each T cell subset clonally related to those present in the circulation designated as mobile. Skin samples with fewer than 6 T cells were excluded from analysis. Values are quantified as a fraction of the clones from each subset. Horizontal bars show the mean frequency of each comparison and frequencies belonging to the same subject are connected with dashed lines. Data for the same *n* = 6 subjects from cohort 1 are shown for all panels except for **B**, which only shows *n* = 4 subjects with B cells that could be traced to the circulation. Statistical differences are shown only when significant for a paired *t* test (***P < 0.001; **P < 0.01; *P < 0.05). EM, erythema migrans; BCR, B cell receptor.

B cell and T cell subsets displayed different levels of connectivity with the circulation (Figure 5). Compared with memory B cells, a higher frequency of plasma cell clones was mobile (paired ratio *t* test *P* = 0.007; Figure 5, A and B). Among T cell subsets, the frequency of mobile Treg clones was less than other T cell subsets (*P* = 0.006, ANOVA; Figure 5D). Tregs were less easily traced to the circulation than dividing T cells and CD8^+^ T cell clones (paired ratio *t* test *P* = 0.006, *P* = 0.003, respectively; Figure 5D); CD4^+^ T cells were also significantly less mobile compared with CD8^+^ T cells (*P* = 0.043; Figure 5, C and D).

We next compared the gene expression pattern of mobile and resident cells in the EM lesion. Mobile CD4^+^ T cells were characterized by upregulation of IL-2RB1 (Supplemental Figure 15, A and D) and TBX21 (https://doi.org/10.5281/zenodo.4711465). CD8^+^ T cells traced to the circulation were characterized by genes associated with CTL effector differentiation like EOMES, GZMK, and NKG7 as well as chemokine genes like CCL5 and CCL4 (Supplemental Figure 15B and https://doi.org/10.5281/zenodo.4711465). They were associated with an IFN γ signaling signature (*P* = 5.1 × 10^–12^; Supplemental Figure 15E). Memory B cells traced to the circulation were enriched for a signature consistent with cell surface interactions at the vascular wall (Supplemental Figure 15, C and F, and https://doi.org/10.5281/zenodo.4711465). No significant differences in gene expression were identified in our analysis of mobile versus resident Tregs or plasma cells. Thus, B cells that have clonal relatives in the circulation were characterized by a trafficking signature, whereas mobile CD4^+^ and CD8^+^ T cells displayed a skew toward effector activation and trafficking.

### EM-associated unmutated IgM memory B cells were activated and part of diversified clones in the circulation.

**Figure 6:**
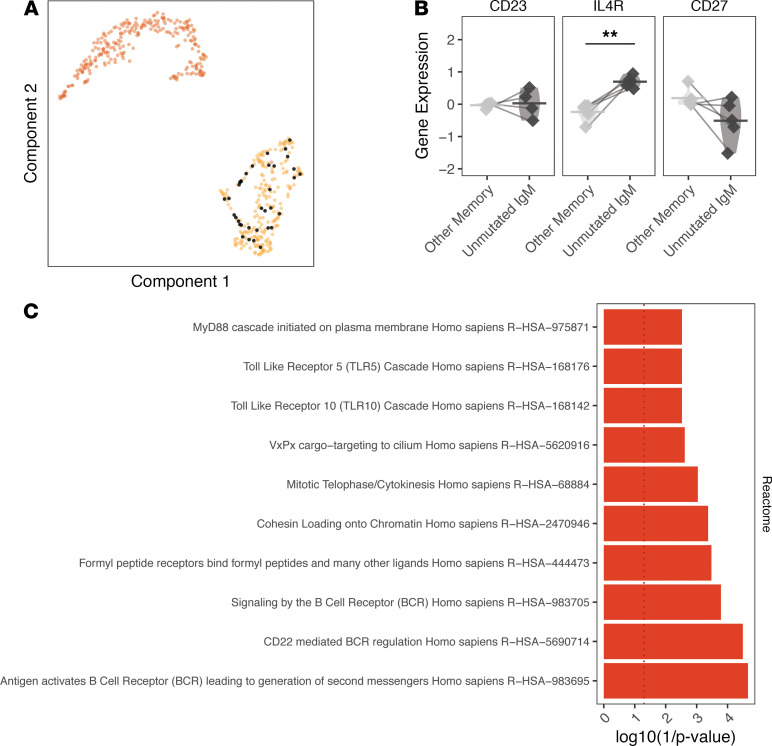
Unmutated IgM/D B cells lack a naive B cell signature. (**A**) Cells with unmutated IgM BCR sequences visualized as black dots over a UMAP projection of B cell single-cell transcriptomes. (**B**) Differential gene expression of 3 marker genes associated with naive B cells after normalization (cell gene expression was *Z*-scored for each grouping and pseudobulk averages were calculated). (**C**) enrichR pathway analysis (Reactome 2016) of the top 40 genes upregulated in unmutated IgM memory B cells compared with all other memory B cells. Red bars correspond to significantly associated gene ontology assignments (*P* < 0.05 by Wilcoxon’s test). Horizontal bars show the mean frequency of each comparison and frequencies belonging to the same subject are connected with lines. Data for the same *n* = 6 subjects from cohort 1 are shown for **A**, while **B** only shows the *n* = 5 subjects with unmutated IgM memory B cells in EM lesions. Statistical differences are shown only when significant for a paired *t* test (**P < 0.01). EM, erythema migrans; BCR, B cell receptor.

Given previous work showing that T-independent B cell production of antibodies can protect against *B*. *burgdorferi* infection (20, 21), we sought to better characterize the phenotype of the unmutated IgM memory B cell subset. We found that unmutated IgM memory B cells, defined as having less than 1% mutation frequency, could not be detected in uninvolved skin from any subject. To confirm that unmutated IgM memory B cells were not misclustered naive B cells, we examined the expression of genes that may distinguish these subsets. We analyzed the entire gene expression profile of the unmutated IgM memory B cell subset (https://doi.org/10.5281/zenodo.4711465) compared with other memory B cells for genes associated with those reported previously for naive B cells (16). We did not find any naive B cell genes among the set of significantly DEGs. For example, among the marker genes CD27, CD23, and IL-4R, only IL-4R was observed to be more highly expressed among unmutated IgM memory B cells compared with other memory B cells, but this was not significant after FDR correction (IL-4R, paired *t* test *P* = 0.005, *P* = 0.053 after FDR correction; Figure 6, A and B). CD27 expression was not downregulated in the unmutated IgM memory B cell subset (*P* = 0.838).

The gene signature associated with the unmutated IgM memory B cells compared with all other memory B cells included signaling by the BCR (*P* = 1.6 × 10^–4^) and antigen-activated BCR (*P* = 2.2 × 10^–5^; Figure 6C). These unmutated IgM memory B cells also were associated with signatures related to the activation of BCR signaling (antigen activates BCR, *P* = 0.03) and TLRs (TLR10 cascade, TLR5 cascade, and MyD88 cascade initiated on plasma membrane). TLR10 was also observed to be the most highly expressed TLR in EM B cells but not in myeloid or dendritic cells (Supplemental Tables 14 and 15). The expression of TLR10 also correlated with the expression of an IFN-induced tryptophan catabolism gene kyureninase (KYNU; *P* < 2.2 × 10^–16^, Spearman’s correlation = 0.49), which has been associated with immunosuppression and previously identified as upregulated in a bulk transcriptome analysis of EM (10). Overall, the gene signatures of unmutated IgM memory B cells in the EM lesion suggested an activated phenotype, and they displayed a skewed VH and JH gene usage, suggesting they were selected by antigen. Compared with circulating unmutated IgM B cells, EM unmutated IgM memory B cells used less IGHV3-23 (paired *t* test *P* = 0.004), IGHV4-59 (*P* = 0.002), and IGHJ6 (*P* = 0.0005); IGHJ6 is a JH gene associated with long CDR3s and self-reactivity in naive B cell subsets (Supplemental Figure 16, A–C). These findings were also confirmed when comparing the EM B cell repertoire with the circulating B cell repertoire that was analyzed at the single-cell level (Supplemental Figure 16, D–F).

Although the unmutated IgM memory B cells were not clonally expanded within the EM lesion, 5 of these cells (2.3% of EM IgM B cells) had clonal relatives in the circulation (Supplemental Figure 17). Of these 5 clones, 4 included IgG-switched relatives in the circulation, 3 of which were also unmutated. Interestingly, although the IgM memory B cells in the EM lesion were unmutated, diversifying mutations were found in clonal relatives from the circulation in 3 of the 5 clones. Thus, unmutated IgM memory B cells, which are found specifically in the EM but not uninvolved skin, appeared to be activated cells that were related to expanded, class-switched, and diversified B cells in the circulation.

## Discussion

The EM lesion results from local skin infection with Ixodes tick–transmitted *B*. *burgdorferi* spirochetes. The act of tick feeding and the associated immunomodulatory effects of tick saliva disrupt normal wound healing and create a protective niche for *B*. *burgdorferi* to establish infection in the blood-meal host skin (22, 23). This introduction of *B*. *burgdorferi* spirochetes at the bite site and their subsequent expansion elicits the first clinical sign of LD, EM, that we sought to elucidate at single-cell resolution.

The EM lesion has historically been characterized through histopathology and IHC as comprising perivascular and interstitial infiltrates dominated by IFN-γ–producing lymphocytes, macrophages, DCs, and occasional plasma cells (3, 8). Our results confirm and extend these earlier findings by providing the first detailed description at the single-cell level of B cells within the EM lesion. The enhanced representation of B cells was found in all Lyme EM samples in comparison with uninvolved skin. These cells expressed high levels of MHC class II and genes associated with antigen presentation and inflammatory activation, and results were validated using 2 independent cohorts and single-cell sequencing approaches. EM B cells comprised both memory B cell and plasma cell populations, with approximately half possessing identifiable circulating relatives. Memory B cells were disproportionately IgM^+^ and displayed lower overall SHM frequencies compared with plasma cells. A large fraction possessed unmutated germline receptors that were associated with a TLR/MyD88 signature. These IgM^+^ B cells also belonged to clones with circulating relatives but displayed a distinct VH gene repertoire compared with relatives in the blood, suggesting active selection for specific BCRs in situ. These results reveal that B cells recruited to the EM lesion may undergo expansion when in contact with antigens present in the skin microenvironment, which may include *B*. *burgdorferi* or tick-associated antigens as well as those of the host microbiome. As *B*. *burgdorferi* components are known to activate MyD88-dependent TLR signaling, these results support the hypothesis that specialized IgM memory B cells may act as early APCs, serving an important and previously uncharacterized role in the adaptive immune response against *B*. *burgdorferi* that arises at its portal of entry in the skin.

The presence of resident plasma cells and CD20^+^ B cells in healthy skin has been observed previously although their functional roles in this tissue remain poorly understood given their extremely low frequency (14). Although B cells were abundant in EM lesions in our study, we found a low frequency of B cells in uninvolved skin, consistent with these previous reports. None of the B cells in uninvolved skin were clonally expanded and only one IgG1-switched plasma cell could be traced to the circulation. By comparison, approximately 40% of B cells in EM lesions could be traced to the circulation, and approximately 80% were memory cells rather than plasma cells, including a plurality that expressed IgM. Previous literature has suggested that infiltrating IgM B cells may be functionally relevant in inflammation, including IgM ASCs in human skin (24, 25); we believe the B cells present in EM lesions may have played a similar role. The overall character of the B cell infiltrate in EM lesions appeared to be highly distinct when compared with the few B cells typically present in uninvolved skin.

The B cells in uninvolved skin expressed lower levels of CD20, reflecting a mainly plasma cell composition. We also directly demonstrated that memory B cells were observed to be more abundant in EM lesions. The memory B cells present in EM have an antigen-presenting signature (vs. circulating plasmablasts, which may also sometimes have an antigen-presenting phenotype) and signatures of IFN and cytokine signaling. Memory B cells, particularly those expressing MHC class II and costimulatory molecules, have previously been shown to be more abundant in animal models of inflammatory disorders at barrier sites, where they exhibit APC function to T cells and mediate local antibody production (14, 24, 26, 27). We also observed that B cells that could be traced to the circulation displayed signatures consistent with trafficking; some B cell clones could be found in both the skin and the circulation, providing supporting evidence that some of these B cells are not tissue resident. Studies have also shown trafficking of memory B cells to the lung in the context of influenza pneumonia; these B cells can transition to produce IgG-switched antibodies that clear infection (28). Our group has observed the trafficking of B cells, including plasmablast and nonplasmablast subsets, in B cell responses to HSV in the female reproductive tract (29). By tracing B cells using their BCRs, this study provides rigorous human data that antigen-presenting memory B cells directly infiltrated the Lyme EM lesion.

VH gene differences indicate a possible enrichment of B cells in the EM lesion with distinct specificities compared with those from the circulation. This suggests that selected B cells enter the EM lesion based on the specificities of their BCRs. Skewing away from VH3 family genes, as found in the study, has also been observed in the context of myasthenia gravis and specific tissue-like memory B cell subsets in multiple sclerosis by our group; recent studies have also observed this skewing in the context of SARS-CoV-2 infection (18, 30, 31). Other studies of autoimmune diseases, including those driven by IFN signaling such as systemic lupus erythematosus, have noted a similar shift in the global BCR repertoire (32–34). A recent study on circulating plasmablast responses in LD has provided parallel evidence that selection for *B*. *burgdorferi* specificity can be detected at the repertoire level although the exact VH genes differentially used by these B cells was not reported (5). In this regard, a European study examining the B cell repertoire in peripheral blood of patients with EM found an overlap between IgM and IgG CDR3 sequences during acute and convalescent infection samples compared with healthy controls, suggesting that B cell immune responses associated with *B*. *burgdorferi* infection may be detectable in the circulating repertoire (35).

Our finding that unmutated IgM-expressing memory B cells appear to be interacting with antigen suggests a potential source for T-independent IgM that may be important for early protective humoral immunity. The identity of these B cells is therefore of particular interest. There is evidence of a role for innate B cells in mice infected with *B*. *burgdorferi*, but whether similar populations contribute to the human response is unknown (20). IgM B cells that are predominant in EM lesions lack the hallmark features of an innate B cell population. Perhaps the most important evidence concerning this possibility is the distinctly non-naive gene expression profile of infiltrating unmutated IgM memory B cells and their expression of TLR gene programs. These B cells also use less JH6, which is consistent with a non-naive origin, and display a signature of prior antigen contact, suggesting an antigen-experienced phenotype. Although some studies have suggested that innate-like B cell populations in humans possess a distinct non-naive (antigen-experienced) phenotype (36, 37), markers previously found to be associated with these subsets were neither detectable (CD43) nor upregulated (CD5 and NOTCH2) in the subset of unmutated IgM memory B cells in our analysis. These B cells may instead represent a potentially extranodal B cell response that may precede a more robust lymph node response, analogous to what has been reported in *B*. *burgdorferi* infection in mice (38), and may be a source of IgM ASCs, a subset that has recently been observed in human skin (25). As discussed above, the observation of a TLR/MyD88 signature among this subset of B cells is consistent with literature suggesting the relevance of T-independent B cells (20, 21) and the pattern recognition system, including on B cells, in optimal serological responses to *B*. *burgdorferi* (39, 40). *B*. *burgdorferi* components that could drive T-independent B cell responses include its abundant lipoproteins that contain the TLR2-stimulating tripalmitoyl-S-glyceryl-cysteine moiety (e.g., in vivo expressed outer surface protein C and VlsE) as well as *B*. *burgdorferi* peptidoglycan (41, 42).

We also showed high levels of TLR10 mRNA in B cells compared with other subsets, including plasmacytoid DCs, on which it is known to be expressed at low levels (43). TLR1 and TLR10 were the most abundant TLR transcripts in B cells, whereas TLR2 and TLR4 transcripts were the most abundant in myeloid cells. TLR10 is functional in humans and possesses both activating and inhibitory signaling potential depending on the cell system examined (44, 45). Influenza virus infection of primary human monocytes in vitro enhances inflammatory cytokine production, whereas anti-TLR10 antibodies can suppress primary human B cell proliferation and production of cytokines and chemokines in response to BCR stimulation conditions mimicking T-independent or T-dependent engagement (46). A modulatory role for TLR10 on B cell function in LD remains to be elucidated in the temporal evolution of the lesion, although evidence exists for its role in controlling immune responses against other pathogens (46, 47). In this regard, we found a correlation between TLR10 expression and expression of KYNU, previously identified as upregulated in a bulk transcriptome analysis of EM and associated with immunosuppression (10).

Our findings related to T cells, particularly the identification of a Th1 signature and evidence of local IFN-γ production associated with CD8^+^ T cell subsets clonally traced to the circulation, are consistent with previous studies implicating these subsets in the control of local infection including against *B*. *burgdorferi* (3, 4, 39). However, we did not identify a notable role for Tregs because they lacked a clear differential gene expression signature in EM compared with uninvolved skin. Tregs did not significantly upregulate IL-10 in EM compared with uninvolved skin, and the expression levels of this transcript in Tregs were not elevated compared with myeloid subsets. These results are in contrast to the model proposed by Marques et al. for a role for expression Tregs based on bulk transcriptomics of EM lesions (10).

A single EM is a recognized early manifestation of LD, but some patients present with multiple EM, indicating disseminated infection. One subject in our cohort presented with multiple EM and some insights could be gleaned from analyzing this sample. The primary EM from this subject displayed the lowest fraction of infiltrating lymphocytes with the fewest cells from the T cell cluster present in the tissue relative to other subjects from the cohort. Although a robust B cell cluster infiltrate was present (second highest for memory B and plasma cells), it was distinct in terms of isotype usage and SHM frequency. The B cells in the EM lesion of this subject possessed the highest fraction of IgG-switched receptors among memory B cells, the highest fraction of IgA-switched receptors among plasma cells, and the highest mean SHM for IgA-switched receptors among plasma cells. IgA-serum reactivity to *B*. *burgdorferi* has been observed in up to a third of subjects with early disease and IgA-positive subjects were more likely to have disseminated disease (48). More extensive studies are warranted to determine whether a reduced T cell response and low frequency of unmutated IgM antigen-presenting memory B cells, as seen in the subject with disseminated disease in our study, represent a suboptimal skin immune response that is permissive to *B*. *burgdorferi* dissemination.

Our findings may have important significance for the study of host defense against infection with *Treponema pallidum*, the spirochetal agent of syphilis, and other epidemic treponematoses that enter through the skin (49). Sexually transmitted *T*. *pallidum* infection gives rise to the primary syphilitic chancre at its portal of entry through mucosal surfaces, especially in oral or genital areas. Histopathology of the lesion in humans reveals abundant T cells and macrophages as well as varying numbers of plasma cells (50, 51). B cells are present but have not been characterized. *T*. *pallidum* differs from *B*. *burgdorferi* in that it has a paucity of outer membrane proteins (52) and a vaccine directed at the rare outer membrane proteins has been elusive. Study of the syphilitic chancre using scRNA-Seq and BCR sequencing may therefore provide insight into the role of B cells in the pathogenesis of syphilis at its earliest stage of infection and aid in vaccine design.

Conclusions from our study may be limited by the modest sample size (*n* = 10) and the predominance of older female subjects. However, we show that our transcriptomics findings were agnostic to the type of scRNA-Seq performed because 2 approaches (10× Genomics and Seq-Well) produced the same qualitative results in terms of differential gene expression for key cell subsets like B cells, CD4^+^ T cells, and CD8^+^ T cells. We did not examine T cells bearing γδ TCR because there was poor recovery of these receptors from repertoire sequencing using the emulsion-based platform. Although comparison was made between autologous uninvolved skin biopsies with lesional skin in our studies, we cannot exclude a potential contribution of cells in the EM biopsies that may be trafficking to the site but still within the vasculature.

In summary, our single-cell analysis supports a role for B cells in antigen presentation and antibody production in the cutaneous response to *B*. *burgdorferi* infection in humans. The finding of unmutated IgM memory B cells in the EM lesion, possibly a consequence of TLR-mediated responses, suggests that these cells could have contributed to early defense against this pathogen prior to a more robust T-dependent response. The expression of TLR10 on these cells could have also contributed to paradoxical local immunosuppression that drives resolution of the lesion in the absence of antimicrobial intervention. Additional studies are warranted to examine the contributions of B cell subpopulations to host defense and resolution of clinical signs like EM.

## Methods

Further information can be found in Supplemental Methods.

### Subject selection.

Ten adult subjects with EM and fulfilling the CDC case definition for LD (10) were recruited from study sites affiliated with the Yale New Haven Hospital in New Haven County, Connecticut, USA, and the Mansfield Family Practice. These subjects made up 2 cohorts (*n* = 6 cohort 1, *n* = 4 cohort 2) enrolled between the months of May and October in 2 consecutive years (2018, 2019). All but 2 subjects (no. 9) tested positive on the Immunetics C6 Lyme EIA (purchased through Thermo Fisher Scientific), which was performed according to the manufacturer’s protocol and interpreted using the company’s recommended Lyme index values of greater than or equal to 1.10 (positive), 0.91–1.09 (equivocal), or less than or equal to 0.90 (negative; ref. 13).

### Sample preparation.

Blood samples and skin punch biopsies of the leading edge of the EM lesion were collected from all 10 subjects, whereas control skin from uninvolved skin at least 2 cm away from the EM leading edge was collected from 7 subjects. PBMCs in whole blood were isolated by Ficoll-Paque PLUS (GE Healthcare) gradient centrifugation. After red blood cell lysis using ACK buffer (Thermo Fisher Scientific, A1049201), cells were washed and resuspended in 90% human sera AB (GeminiBio) and 10% DMSO for cyropreservation for bulk BCR and TCR sequencing. Skin biopsies were obtained with a 3-mM disposable biopsy punch (McKesson and Integra Life Sciences), and samples were placed immediately into MACS Tissue Storage Solution (Miltenyi Biotec, 130-100-008) for transport to the laboratory. Tissues were immediately processed into single-cell suspensions using the Whole Skin Dissociation Kit (Miltenyi Biotec, 130-101-540) according to the manufacturer’s recommendation. Briefly, the tissue was washed in DMEM media (Thermo Fisher Scientific, 11960), placed in the enzyme solution, and incubated in a 37°C water bath for 3 hours. Thereafter, the tissue cells were dissociated using the MACS Dissociator (Miltenyi Biotec, 130-093-235), preprogrammed for skin cell isolation (program h-skin-01). The cells were then washed in DMEM, filtered with a 70-μm filter, pelleted and resuspended in DMEM, and counted. Cells were processed immediately for single-cell library preparation as described below.

### Bulk repertoire preparation, sequencing, and preprocessing.

RNA was prepared from frozen PBMCs using the RNeasy Mini Kit (QIAGEN) per the manufacturer’s instructions. BCR and TCR RNA libraries were prepared using reagents from New England Biolabs as part of the NEBNext Immune Sequencing Kit (a gift of Eileen Dimalanta and Chen Song, New England Biolabs, Ipswich, Massachusetts). These steps were performed as previously described with the inclusion of an additional set of TCRB-specific primers on the 3′ end (53, 54). Libraries were sequenced by 325 cycles for read 1 and 275 cycles for read 2 base-pair paired-end sequencing with a 20% PhiX spike on the Illumina MiSeq platform according to manufacturer’s recommendations. Bulk repertoire data processing and analysis was carried out using tools from the Immcantation framework (http://immcantation.org) as described in ref. 18. Preprocessing was carried out using pRESTO v0.5.11 and the IMGT human germline IGHV reference database (IMGT/GENE-DB v3.1.19; retrieved May 30, 2019) as described in refs. 53, 54. BCR isotypes were assigned by local alignment of the 3’ end of each sequence to a set of constant region sequences. Data were deposited in the NCBI’s Gene Expression Omnibus database (GEO GSE169440).

### Emulsion-based single-cell library preparation and gene expression analysis.

Single-cell preparations were loaded into the Chromium Controller (10× Genomics) for emulsion generation, and libraries were prepared using the Chromium Single Cell 5′ Reagent Kit for version 1 chemistry per the manufacturer’s protocol. Libraries were sequenced on the NovaSeq 6000 with 100 × 100 or 150 × 150 paired-end reads for gene expression and BCR/TCR libraries, respectively. Base calls were converted to fastq sequences and demultiplexed using the cellranger v3.1.0 mkfastq function and aligned to the coding sequences of the GCRhg38 genome supplied by 10× Genomics. Sparse count matrices, barcode assignments, and feature calls were made using the cellranger count subcommand.

An average of 128 million reads were sequenced and an average of around 7000 cells were identified per sample (Supplemental Table 1). The resulting output was loaded into Seurat v.3.1.5 for analysis (55). Cells with fewer than 400 or more than 4000 genes detected or mitochondrial content above 15% of all transcripts were discarded (genes expressed in fewer than 5 cells in all samples were also excluded). A total of 65,363 cells were identified. Single-cell expression values were then scaled using log normalized count values (56). Highly variable genes were identified using FindVariableGenes. The IntegrateData function (using the first 30 dimensions, the default parameter) was used to correct for batch effects. The first 20 dimensions were then selected for clustering analysis based on the location of the inflection point from PCElbowPlot. Clusters from total cells were assigned using the FindClusters function based on a shared nearest neighbor algorithm using a resolution of 0.4 (value at which most major clusters could be resolved; ref. 55). Clusters were annotated based on the expression of a panel of marker genes (Supplemental Figure 3 and Supplemental Table 2). This procedure was repeated for cells from the T cell and B cell clusters that were associated with a TCR or BCR sequence from single-cell repertoire sequencing, respectively. We defined cells as associated with a TCR when repertoire sequencing identified an associated TCRB V(D)J sequence, and as associated with a BCR when the repertoire sequencing identified both IGH and IGK/L sequences. We did not require a paired TCRA because only 60.3% of TCRB were paired with TCRA, given low TCRA expression. In contrast, 96.5% of IGH were found to be paired with IGK/L. To identify clusters, a resolution of 0.7 was used for T cells, whereas a resolution of 0.1 was used for B cells. The Seurat RunUMAP function was run to visualize clusters.

To assign T cell and B cell cluster annotations, cell clusters were first annotated using a “basis set” of published marker genes called immunoStates (Supplemental Table 5 and Supplemental Table 7; ref. 16). The mean expression of each gene (raw transcript counts) was computed for each cluster; each cluster was then assigned to the immunoState subset with the highest Spearman’s correlation coefficient. Tregs and dividing cell immunoStates markers were absent from the immunoStates matrix. These annotations were identified manually using marker genes alone, that is, based on the fraction of each cluster expressing FOXP3 or CDK1 above a specified threshold (Supplemental Figure 6 and Supplemental Table 5). Assignments from the 2 clusters identified from B cell single-cell data were assigned using the immunoStates matrix and then confirmed based on the fractional expression of CD20 and BLIMP1 (Supplemental Figure 4 and Supplemental Table 7).

### Bulk BCR and TCR repertoire V(D)J gene annotation, additional sequence filtering, clonal assignment, germline reconstruction and analysis of SHM, overlap, and phylogenetics.

Reconstructed V(D)J sequences from single-cell sequencing were extracted using the cellranger vdj function from fastq reads. V(D)J germline genes were reassigned using IgBLAST v.1.6.1 also using the September 12, 2018, version of the IMGT gene database for both bulk and single-cell repertoires. After V(D)J annotation, nonfunctional sequences were removed from further analysis for both bulk and single-cell repertoires. For single-cell sequencing–derived repertoires, cells with multiple IGH, TCRB, or TCRA V(D)J sequences were assigned to the most abundant V(D)J sequence by UMI count. In cases where multiple IGH, TCRB, or TCRA V(D)J sequences from the same cell shared the same number of UMI counts, cells were assigned to the most abundant V(D)J sequence by number of paired sequencing reads. V(D)J sequences without matching single-cell transcriptomes were excluded. Any TCRD or TCRG assigned V(D)J sequences from single-cell sequencing was discarded. IGH V(D)J sequences not paired with any matching light chain sequences from single-cell sequencing were also excluded. Bulk and single-cell repertoires were then concatenated and V(D)J sequences were assigned into clonal groups using Change-O v0.4.6 (57). Sequences were first grouped based on IGHV gene, IGHJ gene, and junction lengths. Within these groups, sequences differing by less than a length normalized Hamming distance of 0.18 were defined as clones by single-linkage clustering. This distance threshold was determined based on the average of the local minima between the 2 modes of within-sample bimodal distance-to-nearest histograms using a kernel density estimate as previously described (57). Germline sequences were reconstructed for each clonal cluster with D segment and N/P regions masked (with Ns) using the CreateGermlines.py function within Change-O. To identify TCR clones, clones were assigned if T cells shared the same full-length aligned V(D)J sequence.

To validate the identification of clones based on sharing between bulk and single-cell repertoires, sharing between single-cell repertoires was also quantified for a PBMC and EM sample from subject no. 5. This was done by repeating the same alignment, filtering, and single-linkage clustering approaches described above on these single-cell–only repertoires. In addition, the IGH sequences were then further grouped based on whether the cells shared any common combinations of IGKV with IGKJ and a specific junction length or IGLV with IGLJ and a specific junction length.

Mutations relative to the germline sequence were quantified using the observedMutations function from SHazaM v1.0.1 in R v3.6.1 (58). Clonal overlap was computed using a Bray-Curtis metric implemented using the scipy.spatial.distance.braycurtis function in scipy v1.1.0 (59). Clonal abundance was computed using the estimateAbundance function from Alakazam v1.0.2, with uniform downsampling (to the number of V(D)J from the sample with the fewest sequences to account for different sequencing depth) and 200 replicates of bootstrapping from the inferred complete clonal abundance (18). B cell lineage tree topologies and branch lengths were estimated by maximum parsimony using the dnapars program distributed as part of PHYLIP (v3.697; ref. 60) and the R package dowser v0.0.1. Trees were visualized using the R packages ape v5.4 (61) and ggtree v2.0.4 (62).

To compute background overlap for IGH sequences to justify the presence of significant clonal overlap, clones were reassigned across all subjects using the same clonal threshold of 0.18 described earlier or based on identical V(D)J sequences for TCRB sequences. Bray-Curtis overlap was computed for V(D)J sequences from single-cell–derived IGH repertoires and bulk repertoires from the same subject (“Intra-Subject) or different subjects. The average overlap for comparisons with different subjects was computed (Inter-Subject). A paired 1-tailed *t* test was performed to assess the significance of the log-transformed clonal overlap relative to the background.

### Differential gene expression analysis.

Single-cell log normalized expression values were imputed using the ALRA algorithm to account for dropout during differential gene expression analysis (63). All differential gene expression analyses were performed using pseudobulk averages of gene expression across single-cell transcriptomes; individual gene expression values were first *Z*-score normalized across samples from the same subject before pseudobulk averages were computed (for all differential gene expression analysis; refs. 53, 54). Student’s *t* tests with pairing were performed to assign *P* values for hypothesis testing. FDR correction was performed using Storey’s method implemented within the q value package v2.18.0 in R from Bioconductor after visualization of the *P* value distribution (64). More than 2 cells must be present in each sample to calculate a pseudobulk average for that sample. A *q* value (FDR corrected *P* value) threshold of 0.1 was used for assigning significance for tests of significance from gene expression analysis.

EM compared with uninvolved skin B cell gene expression analysis differed from other calculations in that no paired significance testing was performed given that B cells were identified in only 2 uninvolved skin samples versus all 6 EM skin samples. Therefore, differential expression was computed without paired significance testing or normalization across samples. The minimum absolute differences between EM and uninvolved samples was computed and used to select the top and bottom genes (of note, unpaired significance testing [Student’s *t* test] did not yield significant genes after FDR calculation). A disproportionate fraction of the EM lesion B cell infiltrate in subject no. 6 was observed to be composed of a single expanded, predominantly plasma cell, IgG-switched clone causing the plasma cell fraction of this EM sample to be much higher than that of other samples; at 184 B cell members, this clone was substantially larger than the next largest clone of the data set containing 8 B cell members and was therefore an outlier in terms of size. This clone was excluded from gene expression analysis of this sample to maintain consistent memory B cell and plasma cell ratios across EM samples for gene expression analysis and to reduce noise.

When no significant genes were identified (comparisons of Treg and CD8^+^ T cells from uninvolved and EM skin), the top genes based on absolute difference were analyzed. Gene ontologies were assigned using the enrichr package v2.1 in R, which implements a Wilcoxon’s test for significance using a *P* value threshold of 0.05 for significance (65). A *q* value (corrected *P* value) threshold is reported in cases where this was also less than 0.05. The “Reactome_2016” gene set was used for all pathway enrichment analysis.

### Code and data availability.

Code used in this study will be made available in a publicly accessible repository at https://bitbucket.org/kleinstein/projects/src/master/Jiang2021/ Data for gene expression analysis are available in a publicly accessible repository at https://doi.org/10.5281/zenodo.4711465

### Statistics.

R v3.6.1 was used for all statistical analysis. Dataframe handling and plotting was performed using functions from the tidyverse v1.3.0 in R and pandas v0.24.2 and scipy v1.1.0 in python v3.7.5. All parametric statistical testing (aside from differential gene expression analysis and gene set analysis, described above, was performed in R using the *t* test functions for paired 2-tailed Student’s *t* tests unless otherwise specified. A significance threshold of *P* values of less than 0.05 was used. All tests of the proportions of different cell subsets or frequencies of cells out of 1 were first subjected to a log transformation and are specified by text, i.e., a ratio 2-tailed *t* test was performed. All other tests, including differences in SHM, isotype usage, and gene expression, did not involve a log conversion and are specified by text.

### Study approval.

This research was conducted under human research protocols approved by the IRB of Yale University. Written informed consent was received from all participants prior to inclusion in the study.

## Author contributions

LKB, RRM, DAH, AKS and SHK designed the research studies. LKB, KRD, and AAB participated in subject recruitment and sample collection. All authors participated in data acquisition, analysis and/or interpretation. AAB, KR, and RJ were responsible for the preparation of samples. KBH, HM, RJ, and IF performed bioinformatic analysis. RJ, IF, LKB, and SHK drafted the manuscript, and all authors edited and approved the content for submission.

## Supplementary Material

Supplemental data

## Figures and Tables

**Table 1:**
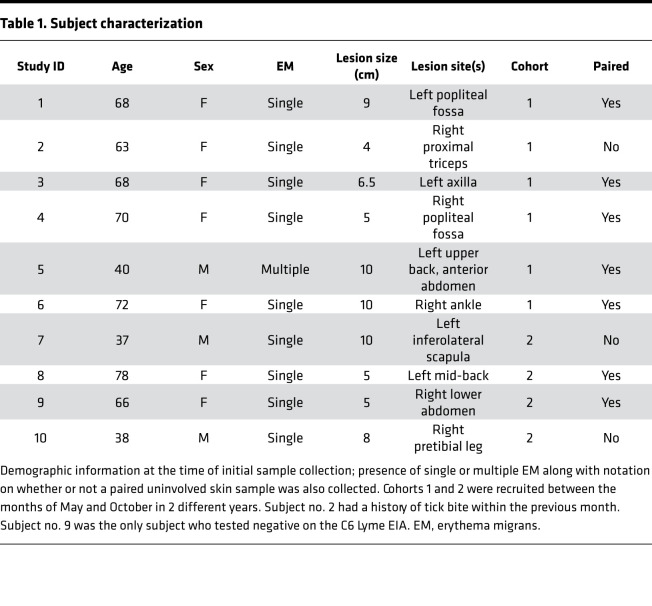
Subject characterization

## Version 1 06/01/2021

In-Press Preview

## Version 2 06/22/2021

Electronic publication
