# Supplementary material for: Single-cell immunophenotyping of the skin lesion erythema migrans identifies IgM memory B cells
Source: JCI Insight. 2021 Jun 22;6(12):e148035. doi: 10.1172/jci.insight.148035 (PMC8262471; doi:10.1172/jci.insight.148035)
Supplement: Supplemental data [file jciinsight-6-148035-s185.pdf]

Supplemental Methods

Seq-Well single-cell library preparation and gene expression analysis

We used the Seq-Well platform as described previously (1). Briefly, 10,000 single cells (200 µl of cell suspensions) were loaded into a Seq-Well array preloaded with barcoded capture beads (ChemGenes). After washing excess beads and cells, the arrays were sealed with a polycarbonate membrane and incubated for 30 minutes at 37ºC. Each cell sequestered in a nanowell with a barcoded capture bead was subject to lysis followed by transcript hybridization. The beads recovered from the entire array were then washed and mixed with reverse transcriptase master mix. The resulting cDNA was amplified using KAPA HiFi PCR Mastermix (Kapa Biosystems KK2602) with 2,000 beads per 50 µL reaction volume. Generated libraries were then pooled and purified using Agencourt AMPure XP beads (Beckman Coulter, A63881) by a 0.6X SPRI followed by a 0.7X SPRI and quantified using nanodrop. Library quality was assessed using a bioanalyzer 2100 (Agilent Technologies).

Single-cell barcoded paired-end sequencing libraries were produced from cDNA with the Nextera XT DNA sample Prep Kit (Illumina #FC-131). Libraries were sequenced on a NovaSeq 6000 sequencer with a 20-50-50 read structure. Base calls were converted to FASTQs using Illumina's bcl2fastq conversion software and reads were aligned to the human hg19 genome using STAR 2.7 (2) alignment software via the Broad Institute's dropseq_workflow pipeline (https://github.com/klarman-cell-observatory/cumulus/blob/master/workflows/drop-seq/dropseq_workflow.wdl), which also converts reads to cell-by-gene sparse count matrices. The output was loaded into Seurat v3.1.5 for analysis. Cells with fewer than 500 transcripts

detected, 300 unique genes detected, or with mitochondrial content above 70% of all transcripts were filtered from the dataset. Integration of assay data was carried out using the Seurat integration pipeline; the first 30 dimensions of canonical correlation analysis (CCA), the default parameter input, were used with the FindIntegrationAnchors and IntegrateData functions. Single-cell expression values were then scaled using log-normalized count values. Cell clusters were assigned using KNN and SNN algorithms using 28 PCs (to contain 99% of the variance in the dataset), a k parameter of $1/2\times\sqrt{}$(total number of cells), and the default resolution of 0.5 (68). The Seurat RunUMAP function was run to visualize these clusters.

Two criteria were used to assign clusters to known cell annotations. (A) Fifty published marker genes for sixteen known skin cell types were used to generate module scores for each cluster using Seurat's AddModuleScore feature (3). (B) The function FindAllMarkers implementing a Wilcoxon test was also used to determine cluster-specific expression; genes specific to each cluster were identified by this method (where $p < 0.01$, log-fold change > 0.25, and at least 10% of cells in the cluster expressed the gene) and ranked by log-fold change. Final cluster annotations were made using these two metrics: module score expression followed by expert correction using differential expression analysis. Of note, a cluster with only mitochondrial top genes (in order of decreasing log-fold change: MT-RNR2, MT-RNR1, MT-ND5, MTRNR2L1, etc.), indicating poor quality, was removed before re-clustering and re-assigning cell types for final annotations.

Average log-fold change was calculated within identified cell types between EM lesion samples and uninvolved skin samples using average expression (FindAllMarkers function) from all

assays pooled for genes where at least 10% of the cells in the annotated cluster are expressed (e.g. 10% of cells expressing a given gene in the identity “EM lesion macrophages”). Correlations between Seq-Well and 10X were computed by identifying the top 20 differentially expressed genes (upregulated and downregulated, a significance filter was first applied only for CD4 T cells) from each subset in comparisons between EM and uninvolved skin. Gene expression differences from emulsion-based single cell transcriptomes using normalized pseudobulk difference were compared against average log-fold change computed values from Seq-Well transcriptomes. Spearman correlation was computed (Figure S8A-D). Of note, the gene expression profile of the myeloid cluster was defined by pooling DC and macrophage clusters (Figure S17).

1. Aicher TP, et al. Seq-Well: A Sample-Efficient, Portable Picowell Platform for Massively Parallel Single-Cell RNA Sequencing. *Methods Mol Biol.* 2019;1979:111-32.
2. Dobin A, et al. STAR: ultrafast universal RNA-seq aligner. *Bioinformatics.* 2013;29(1):15-21.
3. Hughes TK, et al. Highly Efficient, Massively-Parallel Single-Cell RNA-Seq Reveals Cellular States and Molecular Features of Human Skin Pathology. *bioRxiv.* 2019:689273.

Supplemental Figures

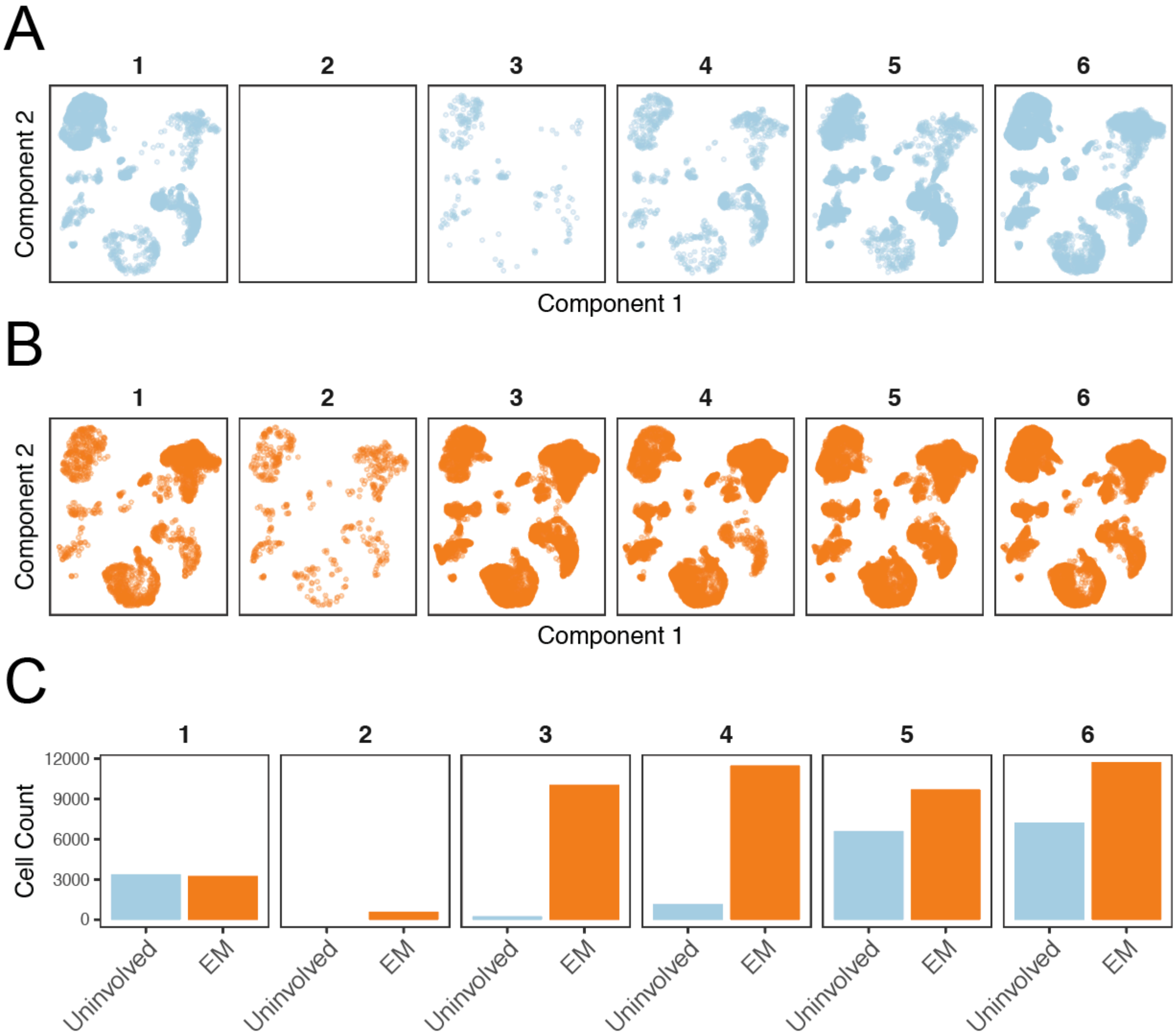

Figure S1. Numbers of cells recovered from each sample and projection of single cell data as UMAP to identify possible batch effects. (A) UMAP projections of total single cell transcriptomes from the sequencing of uninvolved skin per subject. Of note, an uninvolved sample was not obtained or sequenced for subject 2. (B) UMAP projections of total single cell transcriptomes from the sequencing of EM skin per subject. (C) Counts of recovered single cell transcriptomes used in final gene expression analysis after quality control steps from each subject from uninvolved and EM skin samples.

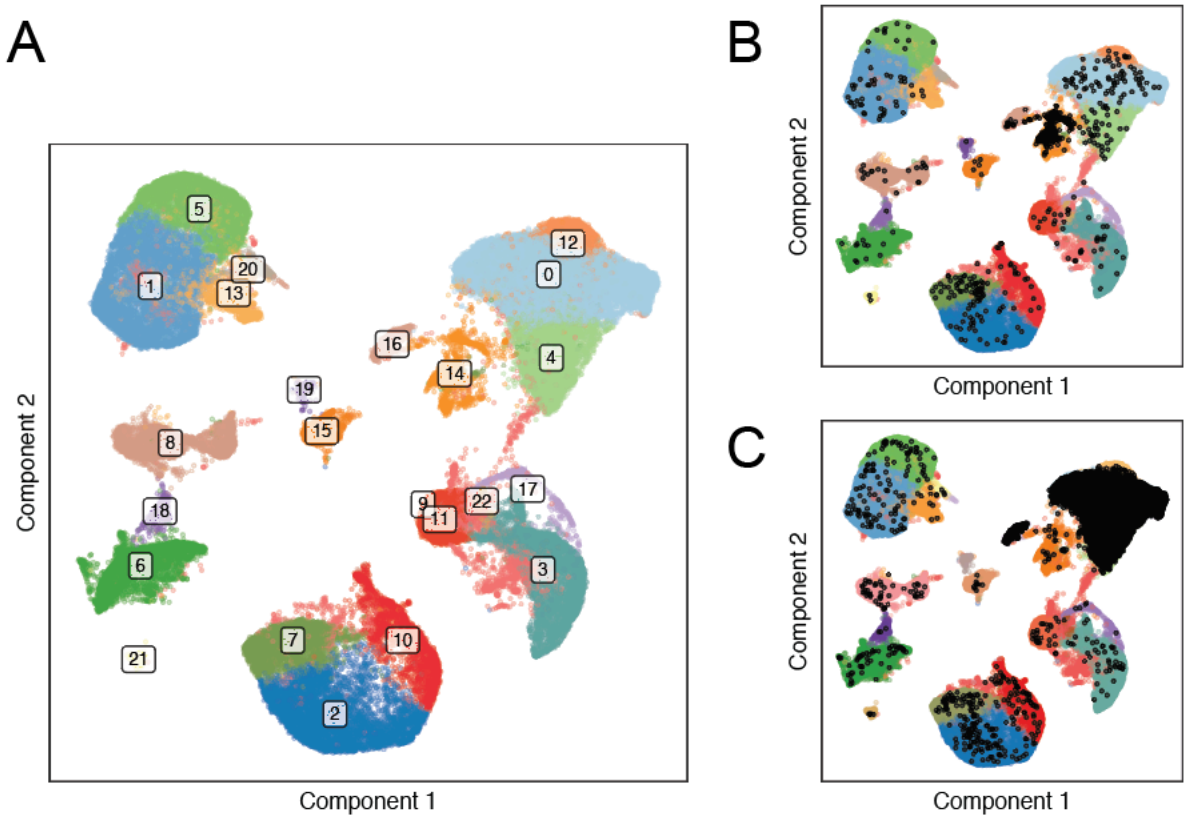

Figure S2. Initial assignment of clusters with overlay of reconstructed BCR and TCR receptors. (A) Clusters assigned by unbiased nearest neighbor clustering algorithm shown on a UMAP projection of total single cell transcriptomes. These clusters are identified using an unbiased algorithm and final assignments are made by analyzing the gene expression profile of the clusters. Corresponding final assignments for clusters can be found in Figure 2A. (B) UMAP projection of total single cell transcriptomes with cells assigned to a BCR receptor with a paired heavy and light chain marked with a black dot. (C) UMAP projection of total single cell transcriptomes with cells assigned to a paired TCRB and TCRA TCR receptor marked with a black dot.

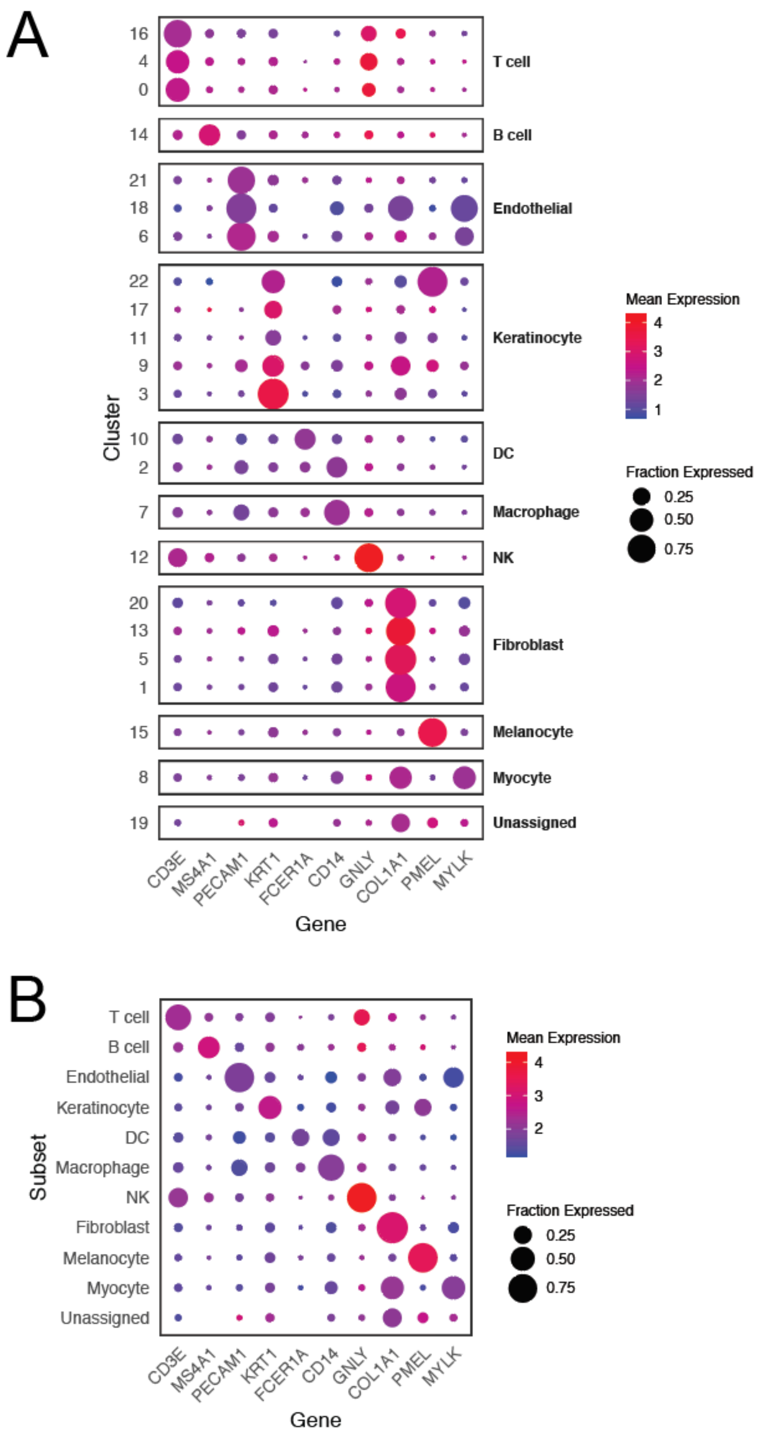

Figure S3. Assignment of clusters from sequencing of Lyme skin infiltrate using marker gene expression. (A) The mean expression and fraction of cells expressing marker genes used for the assignment of clusters to different cell types (raw RNA transcript counts) is shown for each marker gene and each cluster. (B) The mean expression and fraction of cells expressing marker genes is shown for each marker gene and each cluster after assignment.

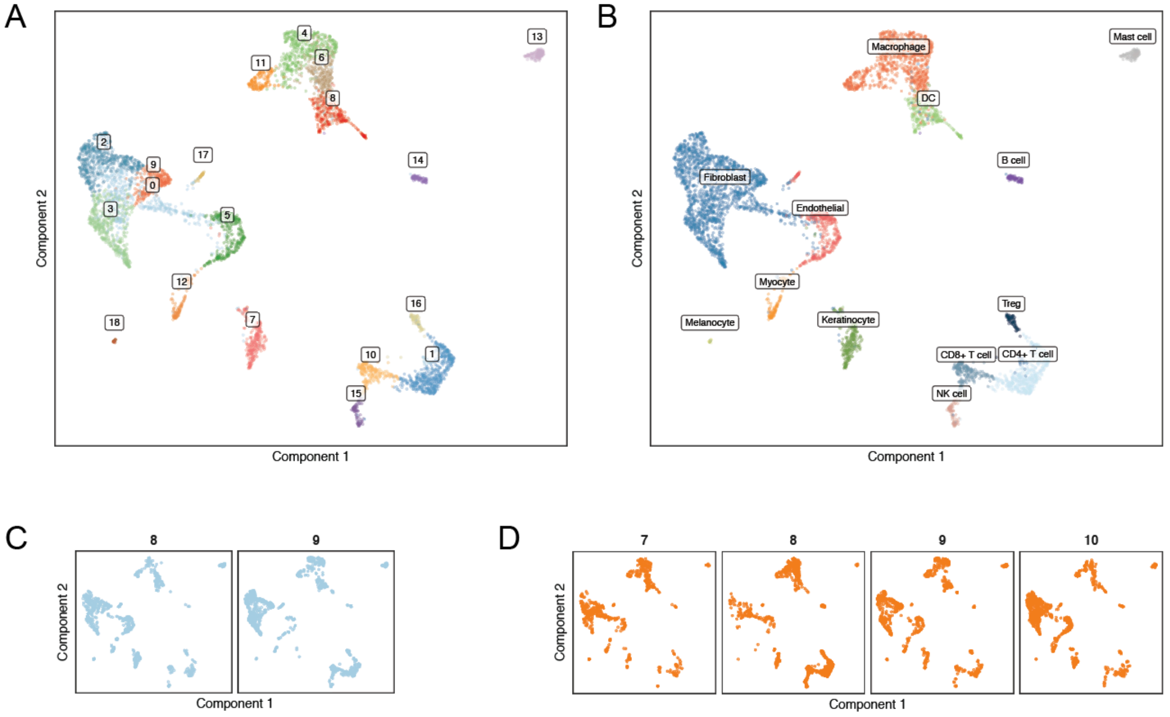

Figure S4. Identification of subsets and assignment of clusters based on marker expression from validation cohort. (A) Clusters assigned by unbiased clustering shown on a UMAP projection of total single cell transcriptomes. These clusters are identified using an unbiased algorithm and final assignments are made by analyzing the gene expression profile of the clusters. (B) Labelled clusters (from analysis of gene expression) are shown on the same UMAP projection. Of note, a "Mast Cell" cluster is shown which was not identified in the Discovery cohort; all other cell types are found using both platforms. (C) UMAP projections of total single cell transcriptomes from the sequencing of uninvolved skin per subject. Of note, uninvolved skin was not obtained for subjects 7 and 10. (D) UMAP projections of total single cell transcriptomes from the sequencing of EM skin per subject.

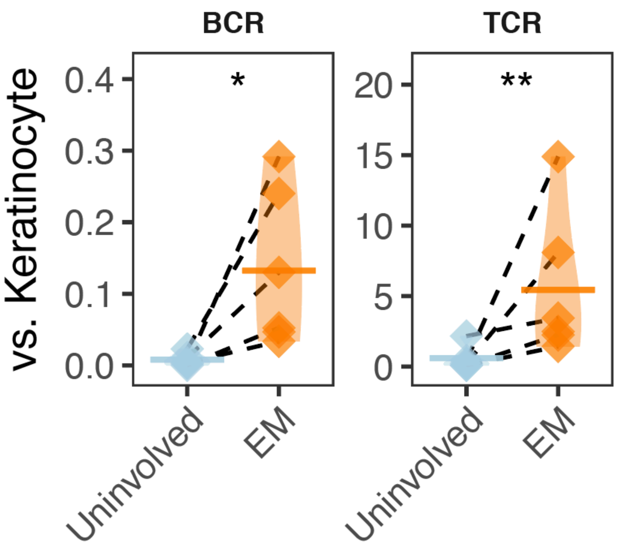

Figure S5. Ratio of cells with paired TCRB or heavy and light chain BCR sequences relative to keratinocytes in EM lesions compared to uninvolved samples. Statistical differences are shown only when significant for a paired ratio t-test (****P < 0.0001; ***P < 0.001; **P < 0.01; *P<0.05).

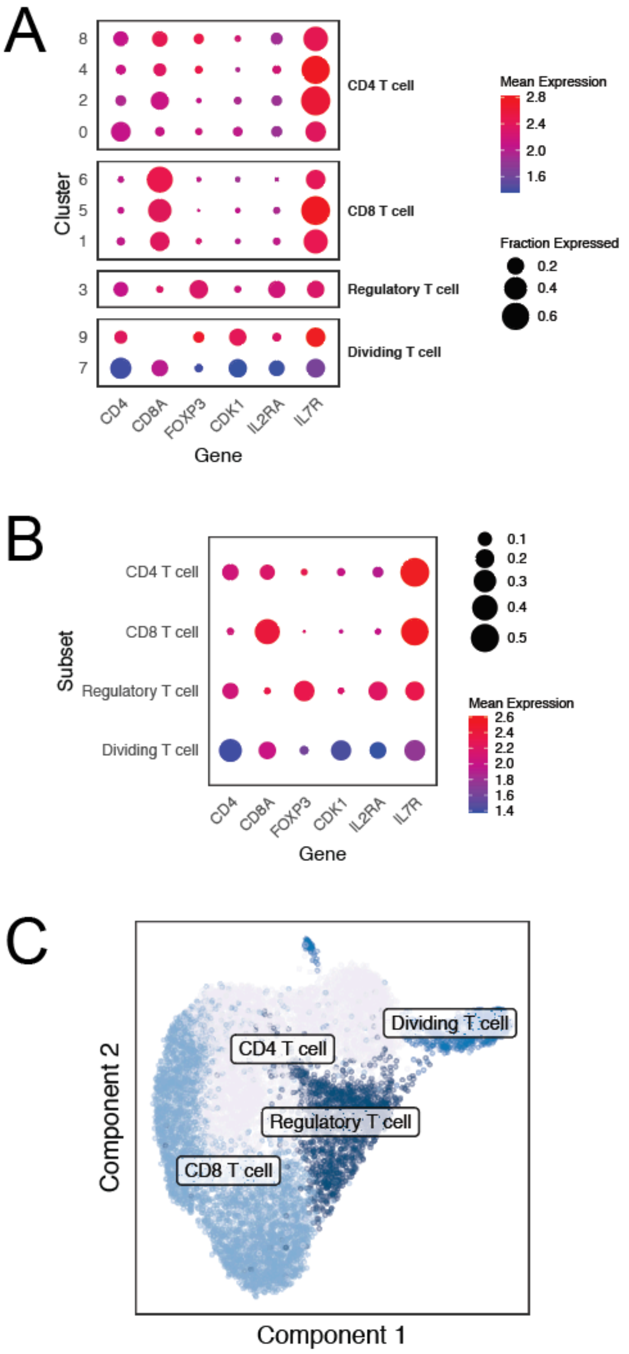

Figure S6. Assignment of T cell clusters (cells must be paired with a TCRB chain) from sequencing of Lyme skin infiltrate using marker gene expression. (A) The mean expression and fraction of cells expressing marker genes used for the assignment of clusters to different cell types (raw RNA transcript counts) is shown for each marker gene and each cluster. (B) The mean expression and fraction of cells expressing marker genes is shown for each marker gene and each cluster after assignment. (C) Labelled clusters shown on UMAP projection of T cell single cell transcriptomes. Colors correspond to different cluster assignments.

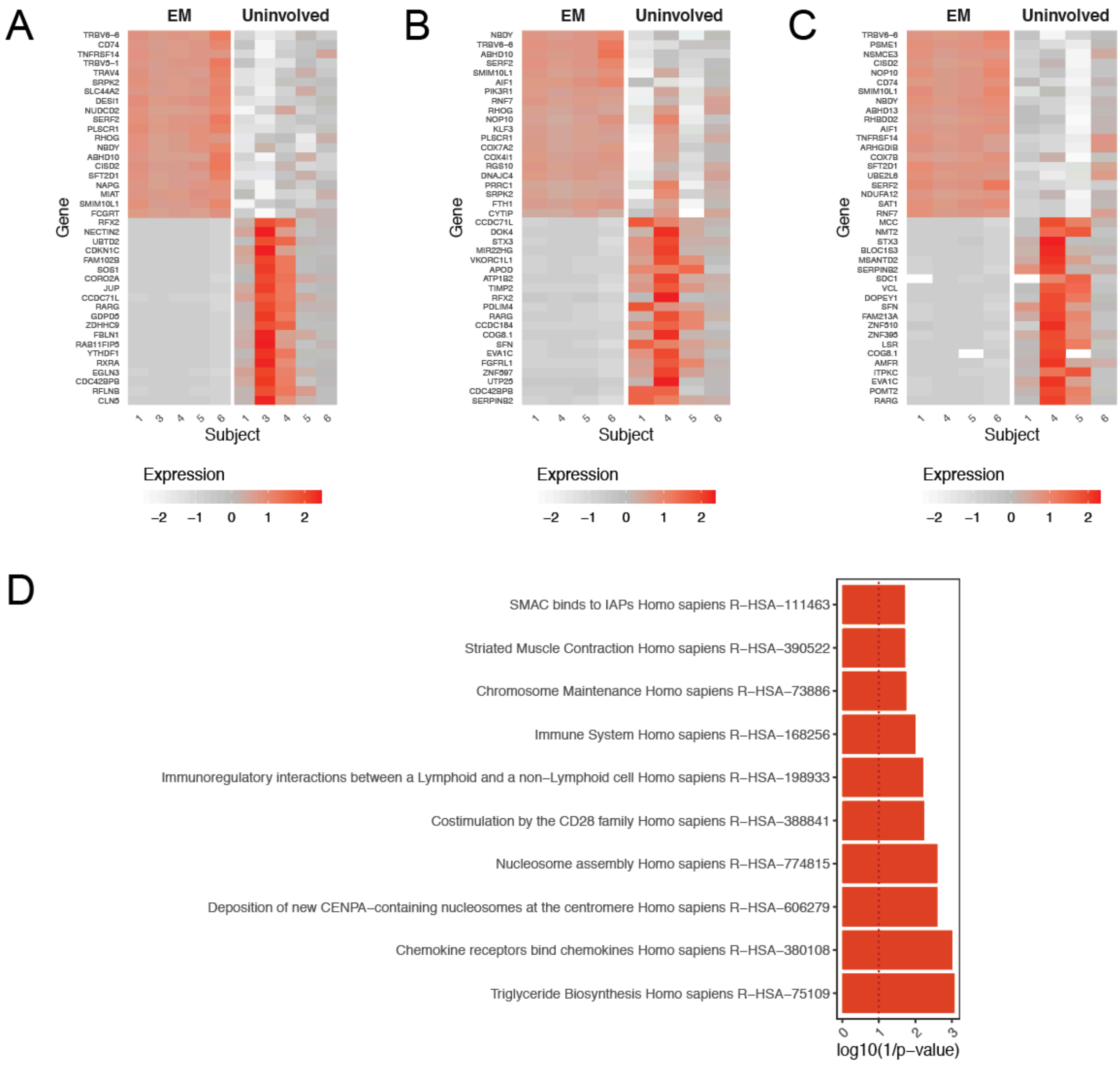

Figure S7. CD4 T cells in EM skin lesion express genes involved in TCR signaling and activation. (A) Heatmap of top 20 upregulated and top 20 downregulated (significantly) differentially expressed genes for CD4 T cells from EM skin compared to CD4 T cells from uninvolved skin ranked based on absolute average gene expression difference. (B) Heatmap of top 20 and bottom 20 differentially expressed genes (by absolute difference) for CD8 T cells from EM skin compared to CD8 T cells from uninvolved skin ranked based on absolute average gene expression difference. (C) Heatmap of top 20 and bottom 20 differentially expressed genes (by absolute difference) for regulatory T cells from EM skin compared to regulatory T cells from uninvolved skin ranked based on absolute average gene expression difference. (D) enrichR gene ontology analysis of the top 40 genes upregulated in CD4 T cells in EM skin compared to CD4 T cells in uninvolved skin. Red bars correspond to significantly associated gene ontology assignments ($p < 0.05$ by Wilcoxon test).

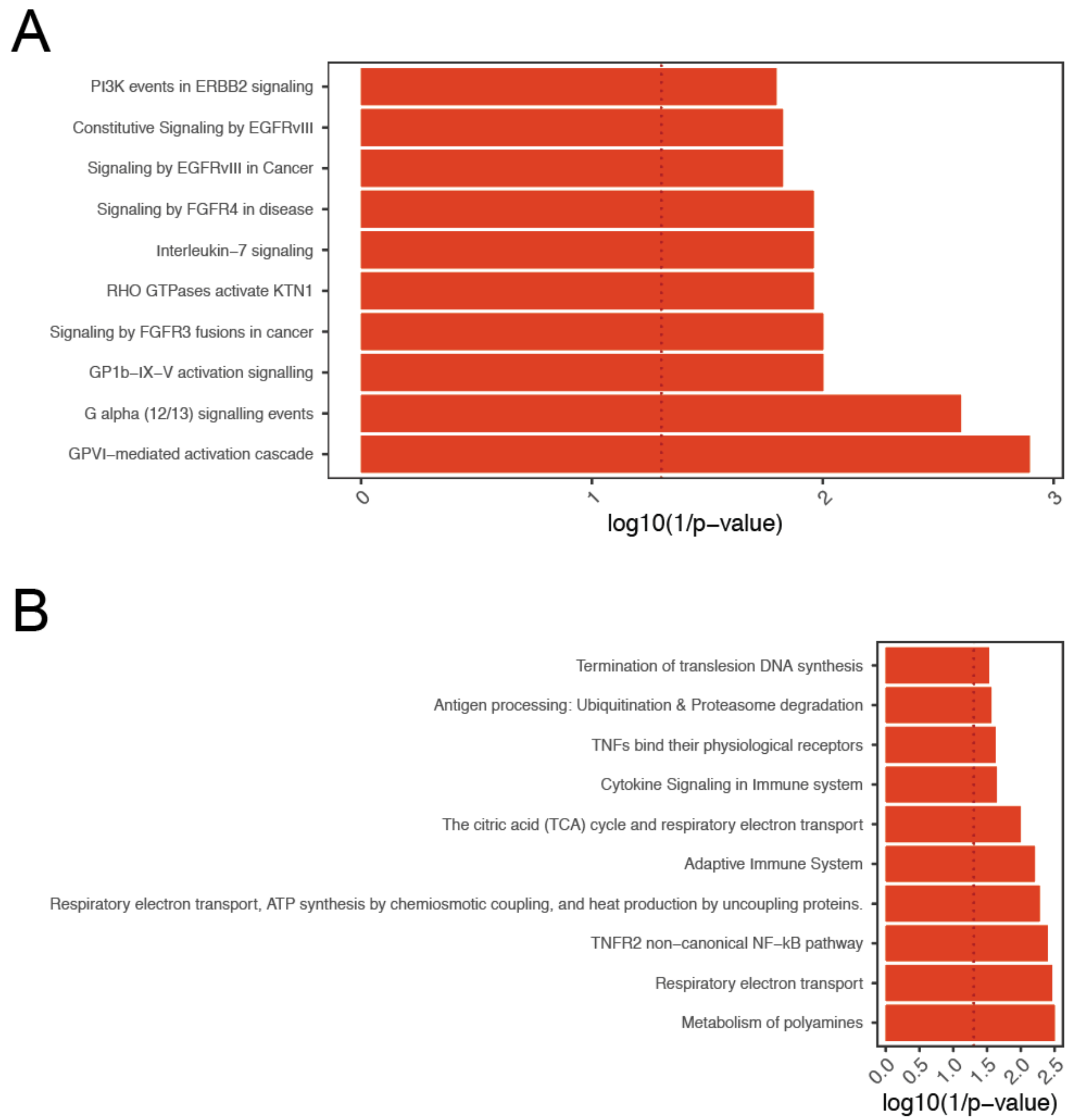

Figure S8. Inflammation related signaling in CD8 T and Treg cells in EM skin. (A) enrichR gene ontology analysis of the top 40 genes upregulated in CD8 T cells in EM skin compared to CD8 T cells in uninvolved skin. (B) enrichR gene ontology analysis of the top 40 genes upregulated in Treg cells in EM skin compared to Treg cells in uninvolved skin. Red bars correspond to significantly associated gene ontology assignments ($p < 0.05$ by Wilcoxon test).

A

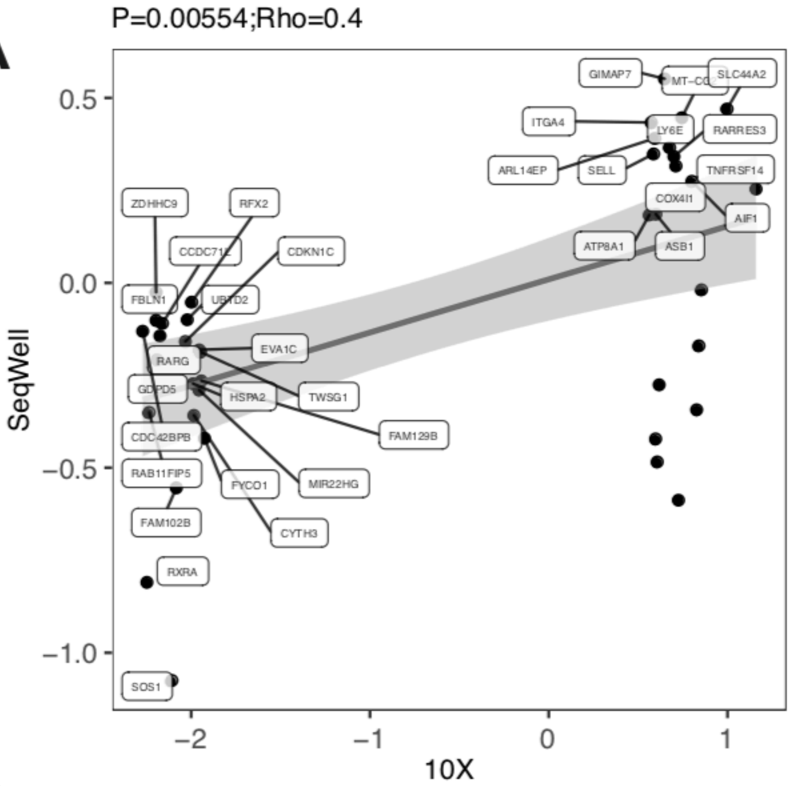
P=0.00554;Rho=0.4
SeqWell
10X
0.5
0.0
−0.5
−1.0
−2
−1
0
1
GIMAP7
SLC44A2
ITGA4
LY6E
RARRES3
ARL14EP
SELL
TNFRSF14
COX4I1
AIF1
ATP8A1
ASB1
ZDHHC9
RFX2
CCDC71L
CDKN1C
FBLN1
UBTD2
EVA1C
RARG
GDPD5
HSPA2
TWSG1
FAM129B
CDC42BPB
RAB11FIP5
FYCO1
MIR22HG
FAM102B
CYTH3
RXRA
SOS1

B

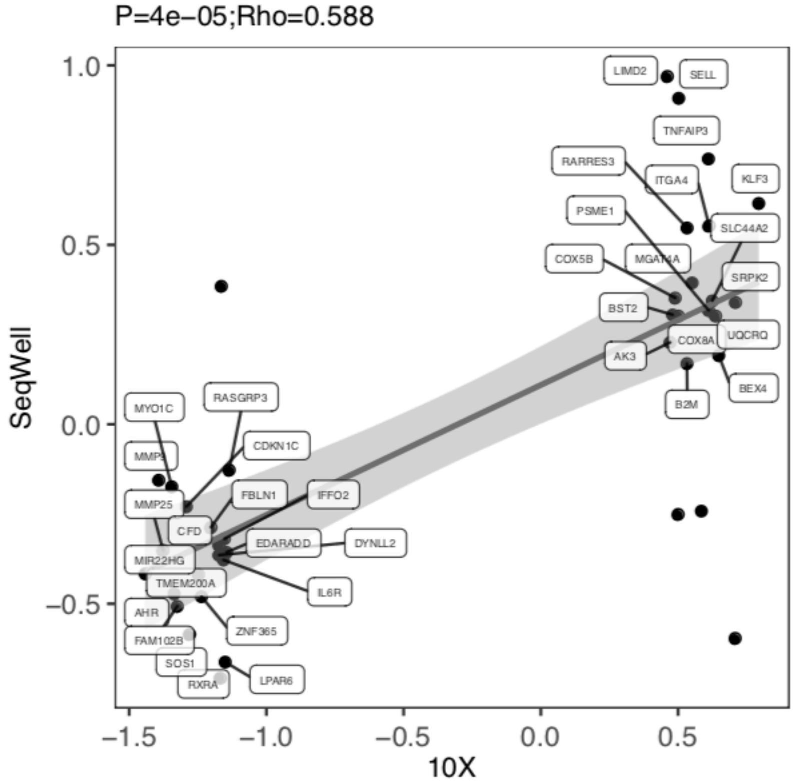
P=4e−05;Rho=0.588
SeqWell
10X
1.0
0.5
0.0
−0.5
−1.5
−1.0
−0.5
0.0
0.5
LIMD2
SELL
TNFAIP3
RARRES3
ITGA4
KLF3
PSME1
SLC44A2
COX5B
SRPK2
BST2
UQCRQ
COX8A
AK3
BEX4
B2M
MYO1C
RASGRP3
CDKN1C
MMP25
FBLN1
IFFO2
CFD
EDARADD
DYNLL2
MIR22HG
TMEM200A
IL6R
AHR
ZNF365
FAM102B
SOS1
LPAR6
RXRA

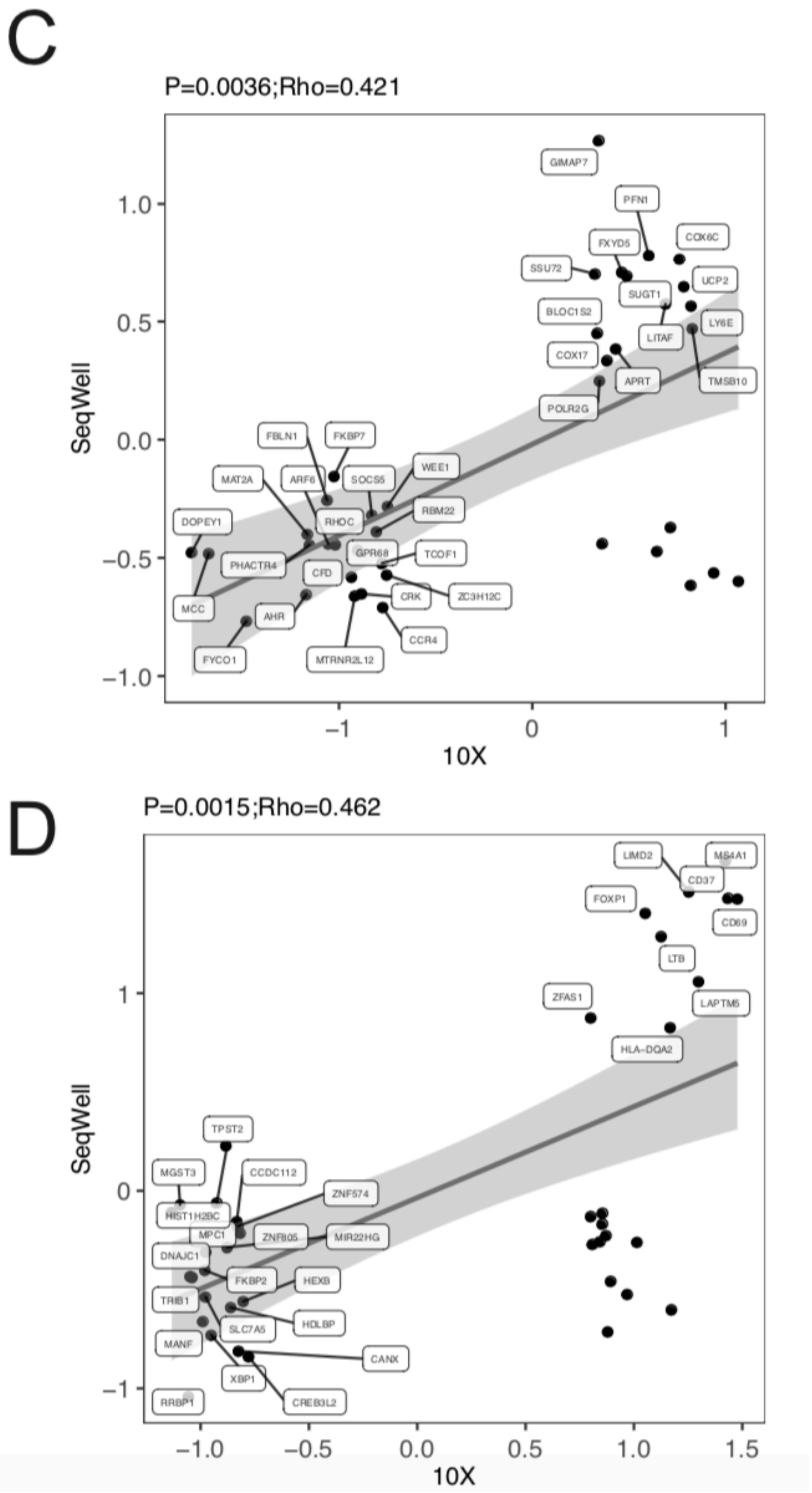
C
P=0.0036;Rho=0.421
SeqWell
10X
GIMAP7
PFN1
FXYD5
COX6C
SSU72
UCP2
SUGT1
BLOC1S2
LY6E
LITAF
COX17
APRT
TMSB10
POLR2G
FBLN1
FKBP7
MAT2A
ARF6
SOCS5
WEE1
DOPEY1
RHOC
RBM22
GPR68
TCOF1
PHACTR4
CFD
MCC
CRK
ZC3H12C
AHR
CCR4
FYCO1
MTRNR2L12
D
P=0.0015;Rho=0.462
LIMD2
MS4A1
CD37
FOXP1
CD69
LTB
ZFAS1
LAPTM5
HLA-DQA2
TPST2
MGST3
CCDC112
ZNF574
HIST1H2BC
MPC1
ZNF805
MIR22HG
DNAJC1
FKBP2
HEXB
TRIB1
HDLBP
SLC7A5
MANF
CANX
XBP1
RRBP1
CREB3L2

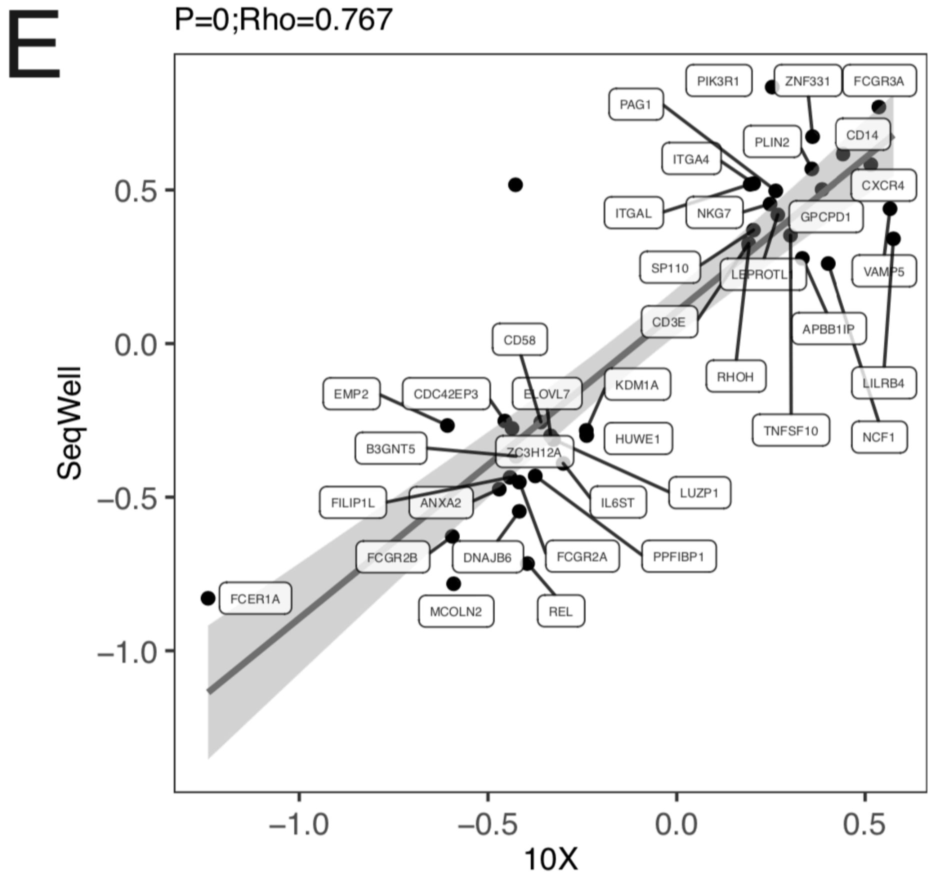

Figure S9. Correlation between EM vs uninvolved skin differential gene expression from two single cell transcriptome platforms for (A) CD4 T cells (top 20 significantly up/downregulated genes), (B) CD8 T cells (top 20 up/downregulated genes), (C) Treg T cells (top 20 up/downregulated genes), (D) B cells (top 20 up/downregulated genes), (E) myeloid cells (top 20 significantly up/downregulated genes), and demonstrating consistent gene expression patterns. Averaged single differential gene expression from pseudobulk gene expression analysis is shown on the x-axis from the discovery cohort while single cell differential gene expression from log fold change expression is shown from the validation cohort on the y-axis if the gene was also detected (if a gene was not also detected by differential gene expression analysis from the validation cohort, it is neither plotted nor analyzed here). Spearman correlation coefficient is shown for each gene along with the P-value for the correlation for each plot.

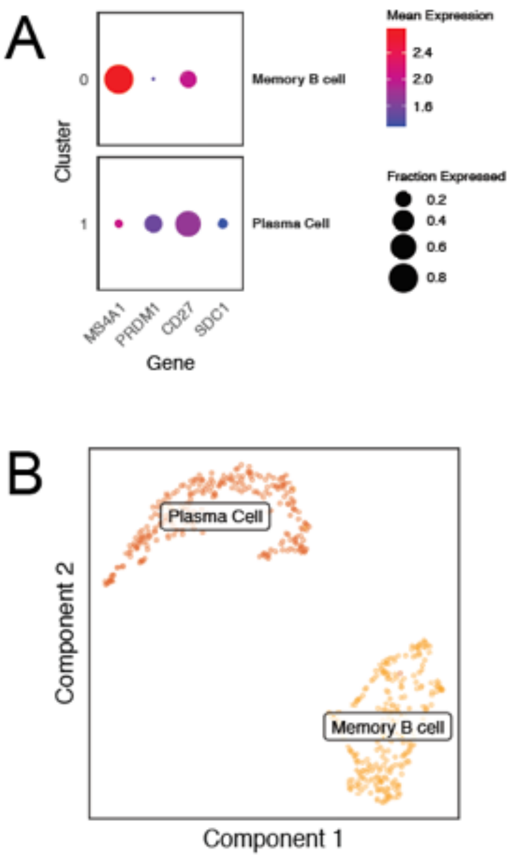

Figure S10. Verification of B cell clusters (and paired with IGH) from sequencing of Lyme skin infiltrate using marker gene expression. Verification of B cell clusters (cells must be paired with a IGH chain) from sequencing of Lyme skin infiltrate using marker gene expression. (A) The mean expression and fraction of cells expressing marker genes is shown for each marker gene and each of the two clusters after assignment. (B) Labelled clusters shown on UMAP projection of B cell single cell transcriptomes. Colors correspond to different cluster assignments.

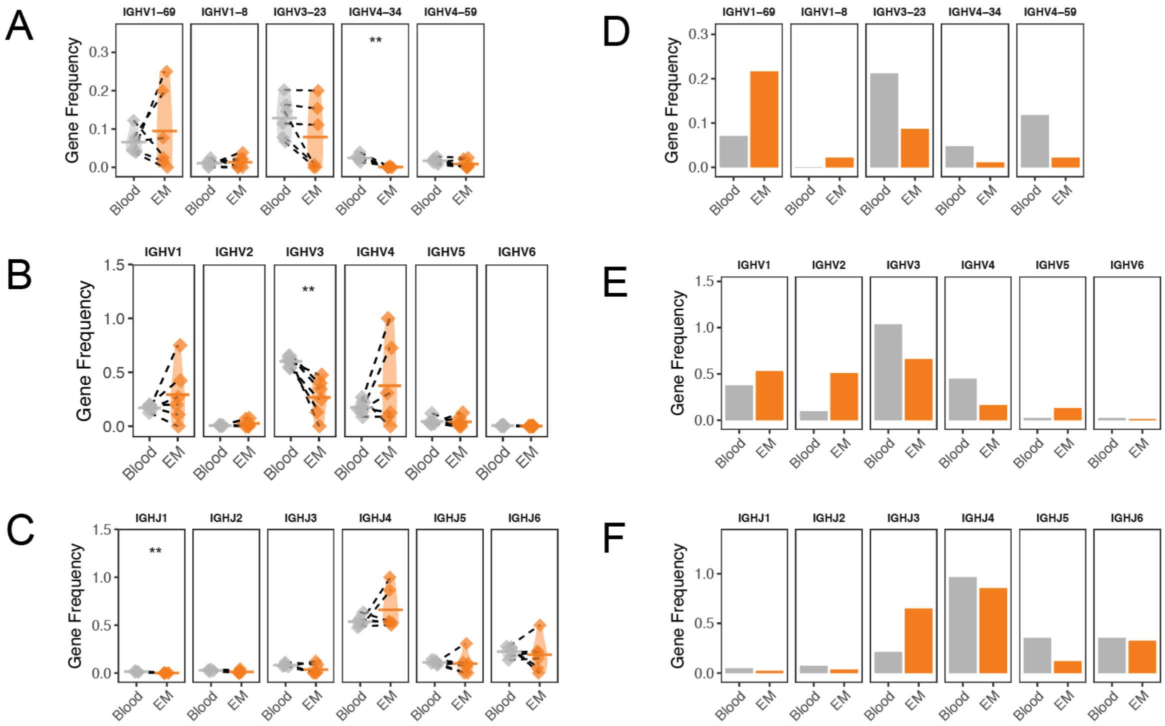

Figure S11. Antigen-experienced (IgA, IgG) B cells present in EM lesions use VH3 family genes less frequently. (A) VH family, (B) VH gene and (C) JH gene usage differences of EM single cell B cell repertoires compared with bulk circulating B cell repertoires for IgG and IgA-switched V(D)J sequences. This analysis is also shown for a single cell EM sample with a paired single cell derived PBMC sample for confirmation(D,E,F). Horizontal bars show the mean frequency of each comparison and frequencies belonging to the same patient are connected with lines. Statistical differences are shown only when significant for a paired t-test (****P < 0.0001; ***P < 0.001; **P < 0.01; *P<0.05).

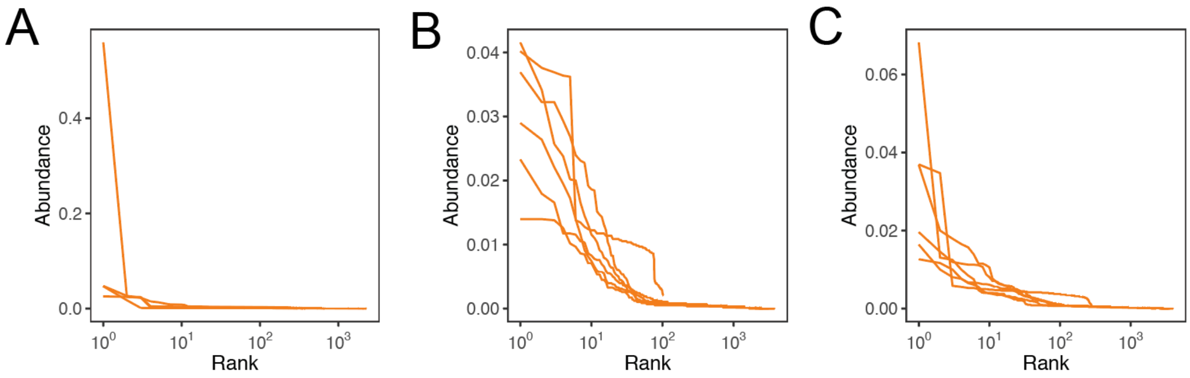

Figure S12. B cell, CD4 T cell and CD8 T cell clonal expansions in EM skin. Rank abundance plots are shown for (A) B cell clones, T cell clones in the (B) CD8 T cell cluster and also T cell clones in the (C) CD4 T cell cluster. Abundance calculations were computed after 200 iterations of bootstrapping with rarefaction from a Chao-estimator corrected distribution.

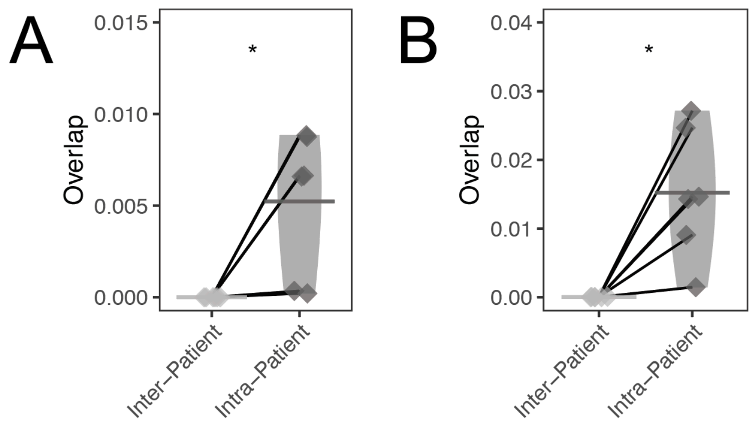

Figure S13. Significant overlap can be identified in EM lesion skin with the circulation among B and T cells. Clonal sharing is quantified using Bray-Curtis overlap. This is shown for (A) B cells and (B) T cells for the sharing of clonal clusters between the skin and circulation of the same individual (intra-individual) and that across individuals (inter-individual). Quantification of clonal sharing from the same patient is paired with a line. Statistical differences are shown only when significant for a paired t-test after natural log transformation (****P < 0.0001; ***P < 0.001; **P < 0.01; *P<0.05).

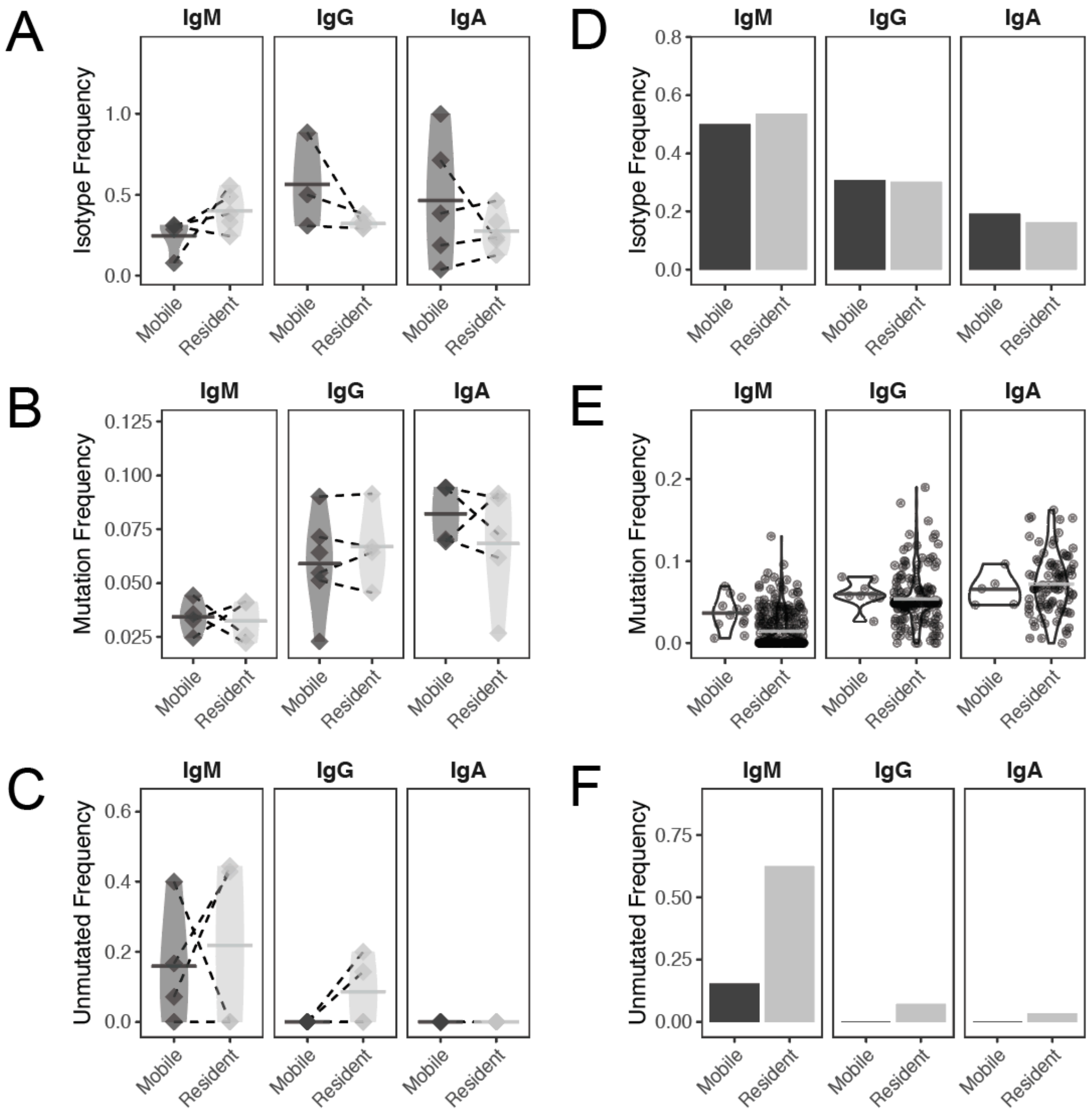

Figure S14. B cells that overlap are similar to their resident counterparts in terms of repertoire features. Potentially more unmutated IgGs are resident. (A) Usage of different isotypes among mobile (shared with circulation) and resident clones is shown for IgM, IgG and IgA in EM skin. (B) Average SHM frequencies among mobile and resident clones are shown for IgM, IgG and IgA in EM skin. (C) Frequency of cells with unmutated V(D)J sequences for IgM, IgG and IgA. Analysis of isotype usage (D), mean SHM frequency (E) and frequency of unmutated sequences for each isotype (F) is also shown for a pair of samples for which the circulating repertoire was also obtained by single cell sequencing. Horizontal bars show the mean frequency of each comparison and frequencies belonging to the same patient are connected with lines. Statistical differences are shown only when significant for paired t-tests (****P < 0.0001; ***P < 0.001; **P < 0.01; *P<0.05).

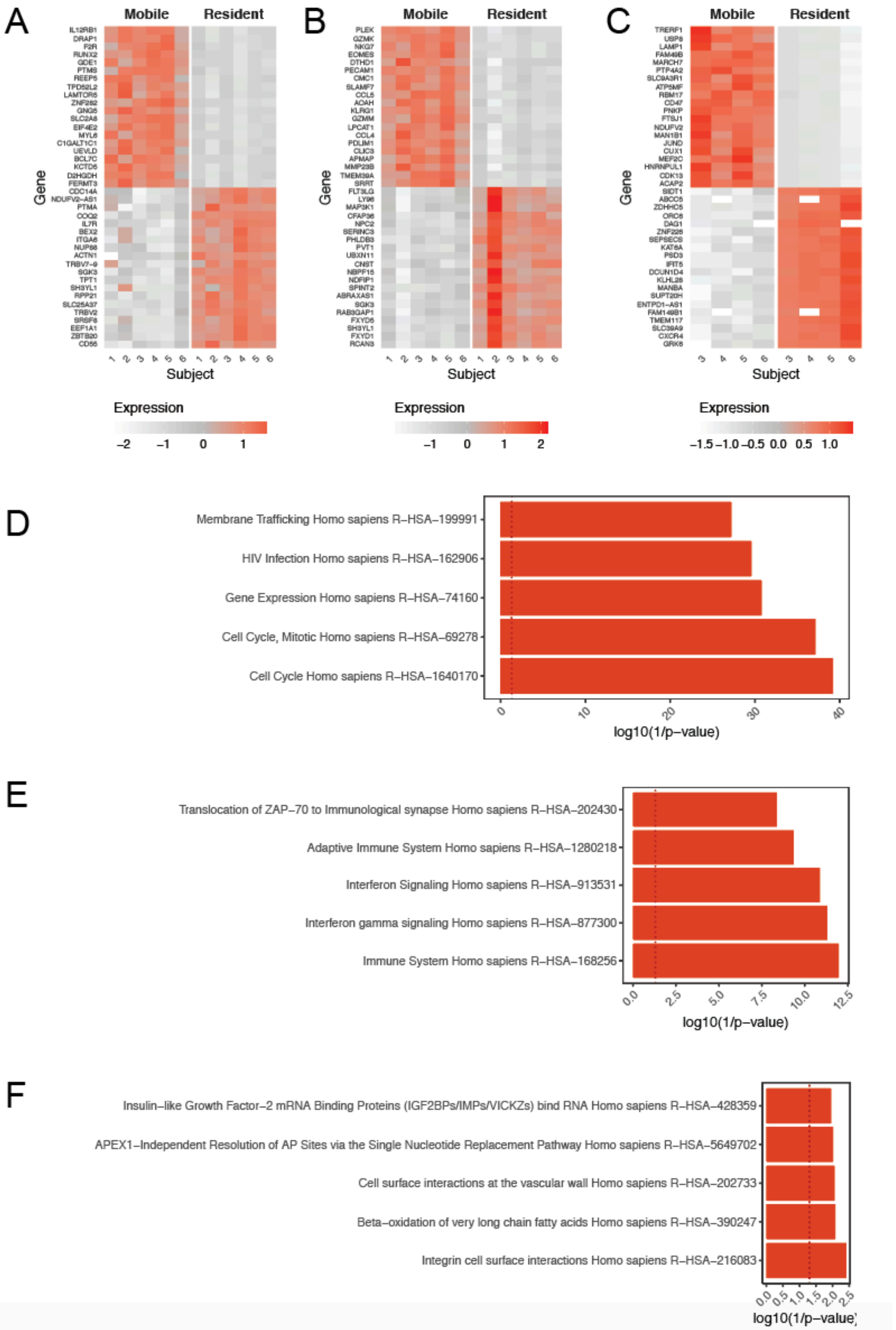

Figure S15. Gene expression signature of emigrant B cells and T cells: T cells have an activation signature and B cells have a trafficking signature. (A) Heatmap of top 20 and bottom 20 significantly differentially expressed genes for CD4 T cells from EM that are found only in the skin compared to those that are also found in the circulation ranked based on absolute average gene expression difference. (B) Heatmap of top 20 and bottom 20 significantly differentially

expressed genes for CD8 T cells from EM that are found only in the skin compared to those that are also found in the circulation ranked based on absolute average gene expression difference. (C) Heatmap of top 20 and bottom 20 significantly differentially expressed genes for memory B cells from EM that are found only in the skin compared to those that are also found in the circulation ranked based on absolute average gene expression difference. (D) enrichR gene ontology analysis of the top 40 genes upregulated CD4 T cells that can be traced to the circulation compared to memory B cells found only in Lyme EM skin. (E) enrichR gene ontology analysis of the top 40 genes upregulated CD8 T cells that can be traced to the circulation compared to CD8 T cells found only in Lyme EM skin (F) enrichR gene ontology analysis of the top 40 genes upregulated in memory B cells that can be traced to the circulation compared to memory B cells found only in Lyme EM skin. Red bars correspond to significantly associated gene ontology assignments ($p < 0.05$ by Wilcoxon test).

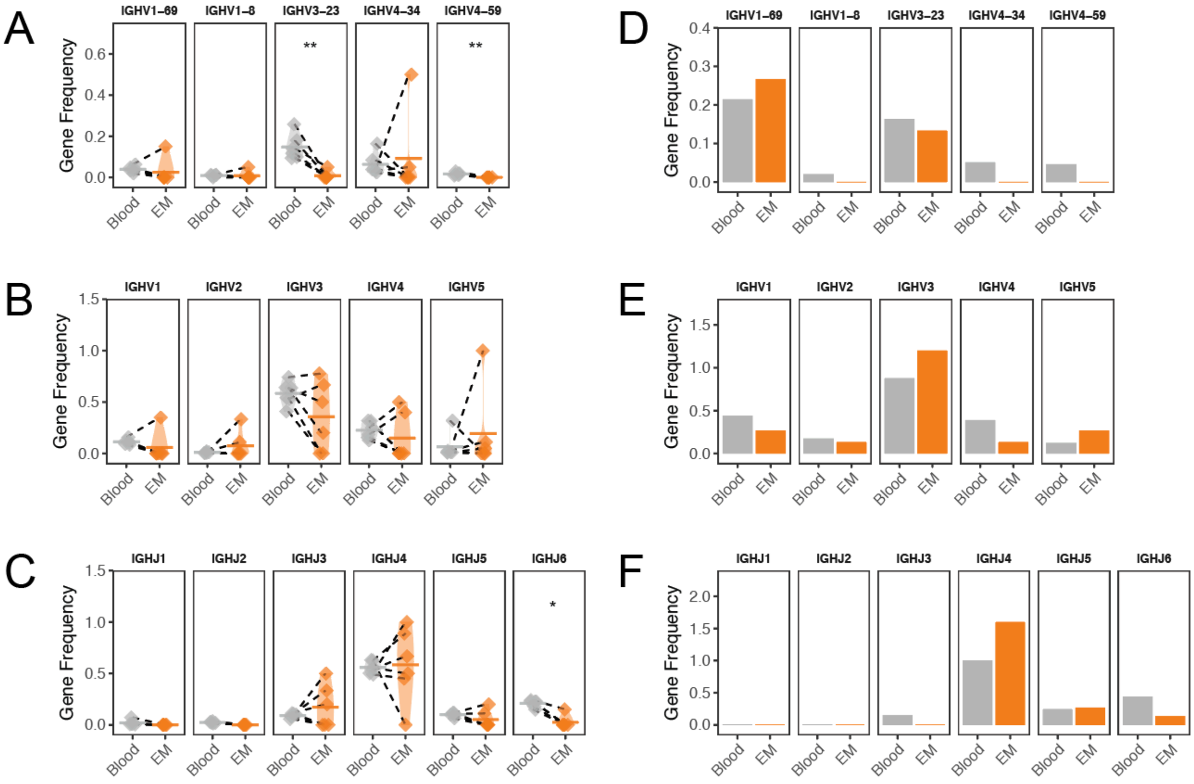

Figure S16. Repertoire features of unmutated IgM/D B cells in EM skin compared to circulation showing reduced usage of IGHV3-23 and IGHJ6. (A) VH family, (B) VH gene and (C) JH gene usage differences of EM single cell B cell repertoires compared with bulk circulating B cell repertoires for unmutated IgM and IgD-switched V(D)J sequences. This analysis is also shown for a single cell EM sample with a paired single cell derived PBMC sample for confirming findings (D, E, F). Horizontal bars show the mean frequency of each comparison and frequencies belonging to the same patient are connected with dashed lines. Statistical differences are shown only when significant by paired t-test (****P < 0.0001; ***P < 0.001; **P < 0.01; *P<0.05).

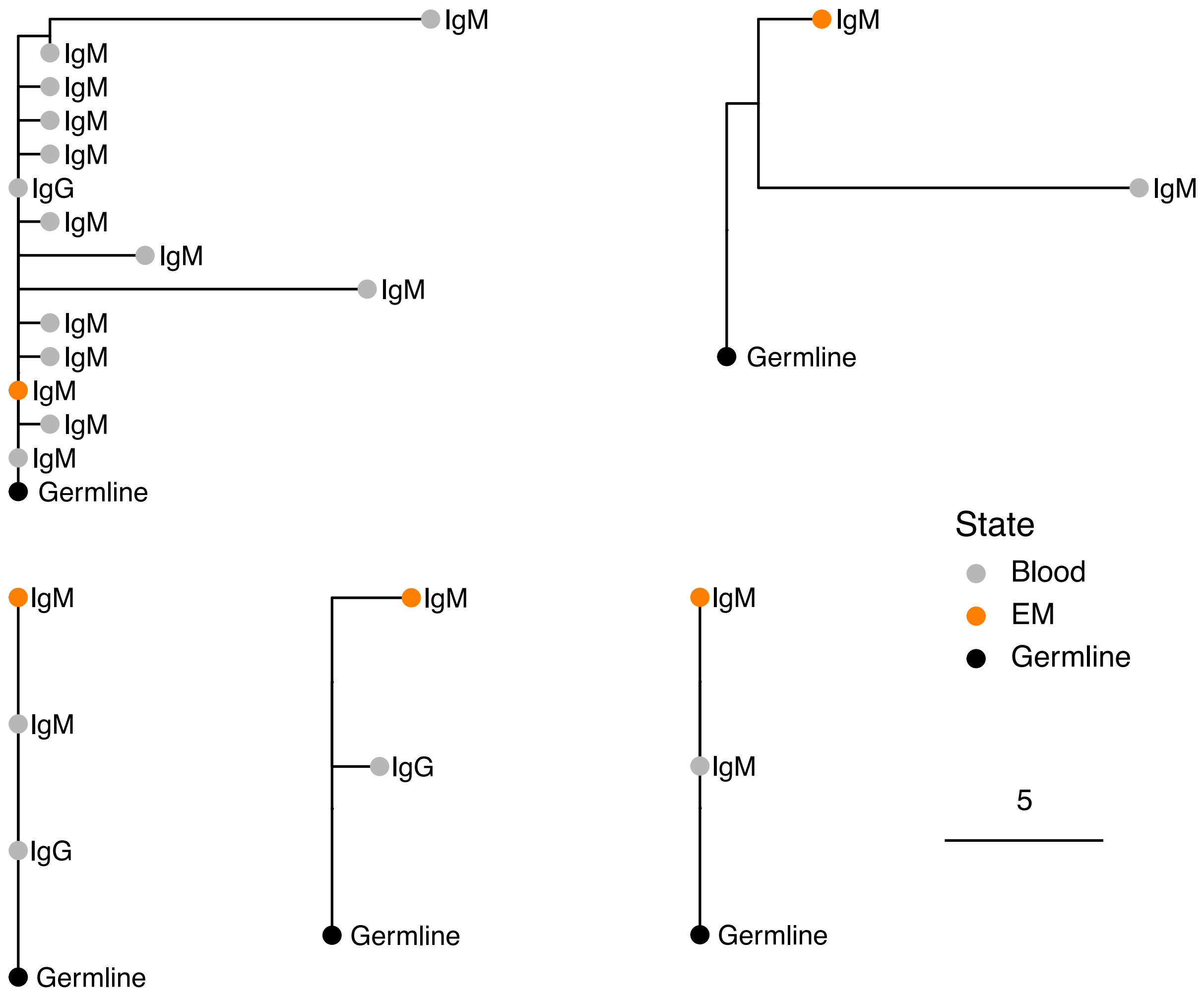

Figure S17. Trees of unmutated IgM B cells also found in the circulation are reconstructed using maximum parsimony. Edge lengths are quantified based on absolute number of intervening somatic hypermutations between observed V(D)J sequences per the scale going from left to right. Colors correspond to whether each V(D)J sequence was collected from a Blood or EM sample (or Germline). The associated constant region assignment accompanies each node as a label.

Supplemental Tables

Patient	Status	Read Count	Cell Count	Mean Reads per Cell	Median Genes per Cell	BCR VDJ Count	TCRB VDJ Count
1	Uninvolved	113339435	4071	27840	1418	3	110
1	EM	121267796	3993	30370	1140	8	1149
2	EM	122987280	695	176960	1355	3	98
3	Uninvolved	131206875	332	395201	1368	0	2
3	EM	132585697	11864	11175	754	78	2196
4	Uninvolved	113627915	1406	80816	1431	1	99
4	EM	132605940	14002	9470	599	118	4110
5	Uninvolved	124527333	10735	11600	505	27	124
5	EM	163662504	10986	14897	1046	202	1681
6	Uninvolved	131237160	7879	16656	1083	19	989
6	EM	123425710	14872	8299	621	234	2263

Table S1. Numbers of reads for gene expression analysis and reconstructed V(D)J sequences from single cell RNA sequencing using an emulsion-based method. Values correspond to the default quality control output from the cellranger count and vdj functions.

CELL	GENE	FRACTION EXPRESSED THRESHOLD	PRIORITY
T cell	CD3E	0.5	1
B cell	MS4A1	0.3	1
Endothelial cells	PECAM1	0.5	1
Keratinocyte	KRT1	0.1	1
DC	FCER1A	0.05	1
Macrophage	CD14	0.5	1
NK cell	GNLY	0.5	1
Fibroblast	COL1A1	0.8	1
Melanocyte	PMEL	0.8	2
Myocyte	MLYK	0.4	2

Table S2. Cell type markers used for assigning each cluster from total single cell RNA sequencing data and the threshold percent of total cells expressing the gene used to assign a cluster to a particular cell type. If a cluster is assigned to multiple cell types, the cell type with the higher priority (1 over 2) is assigned to the cluster (e.g. cluster 22 is assigned to keratinocytes despite also having elevated PMEL expression). Any cell clusters not assigned using this criterion were regarded as Unassigned and removed from further plotting and analysis (cluster 19).

Patient	Status	Read Count	Cell Count	Mean Reads per Cell	Median Genes per Cell

7	EM	25513573	959	26604	687
8	Uninvolved	58866915	1190	49468	1026
8	EM	72010090	1306	55138	1946
9	Uninvolved	81361638	1094	74371	2850
9	EM	79946059	1131	70686	2302
10	EM	22461551	1592	14109	765

Table S3. Numbers of reads from single cell RNA sequencing of validation cohort using microfluidic single cell transcriptome technology.

Cluster	Immunostates	Spearman Correlation	Final Assignment
0	CD4_positive_alpha_beta_T_cell	0.780	CD4 T cell
1	CD8_positive_alpha_beta_T_cell	0.736	CD8 T cell
2	CD4_positive_alpha_beta_T_cell	0.716	CD4 T cell
3	CD4_positive_alpha_beta_T_cell	0.711	Regulatory T cell
4	CD4_positive_alpha_beta_T_cell	0.757	CD4 T cell
5	CD8_positive_alpha_beta_T_cell	0.737	CD8 T cell
6	CD8_positive_alpha_beta_T_cell	0.735	CD8 T cell
7	CD4_positive_alpha_beta_T_cell	0.772	Dividing T cell
8	CD4_positive_alpha_beta_T_cell	0.530	CD4 T cell
9	CD4_positive_alpha_beta_T_cell	0.538	Dividing T cell

Table S4. Table of T cell cluster assignments using the immunoStates basis set. Clusters were assigned to the immunoState with the maximum Spearman correlation coefficient when compared with the mean expression value of genes associated with each cluster. Two CD4 T cell clusters (cluster 7 and 9) were assigned to a dividing T cell subset and another CD4 T cell cluster (cluster 3) was assigned to a regulatory T cell subset based on marker expression (Table S6).

CELL	GENE	FRACTION EXPRESSED THRESHOLD	PRIORITY
CD4 T cell	*CD4*	*0.04*	*2*
CD8 T cell	*CD8A*	*0.25*	*1*
Regulatory T cell	FOXP3	0.2	1
Dividing T cell	CDK1	0.2	1

Table S5. T cell subset markers used for assigning (not italics) or verifying (italics) each subset from total single cell sequencing data and the threshold of expression used to assign a cluster to a particular cell type. If a cluster is assigned to multiple cell types, the cell type with the higher priority (1 over 2) is assigned to the cluster (e.g. cluster 3 is assigned to Regulatory T cells despite also having elevated CD4 expression).

Cluster	Immunostates	Spearman Correlation	Final Assignment
0	memory_B_cell	0.7237	Memory B cell
1	plasma_cell	0.6049	Plasma Cell

Table S6. Table of B cell cluster assignments using the immunoStates basis set. Clusters were assigned to the B cell immunoState with the maximum Spearman correlation coefficient when compared with the mean expression value of genes associated with each cluster.

CELL	GENE	FRACTION EXPRESSED THRESHOLD
Plasma Cell	PRDM1	0.1
Memory B cell	MS4A1	0.5

Table S7. B cell type markers used for verifying each subset from total single cell sequencing data and the threshold of expression used to assign a cluster to a particular cell type.

Clone ID	Cell Count	Unique Sequences	Subset	Constant Region	VH Gene	Junction Length	Overlap
2-3608	1	1	Memory B cell	IGHD	IGHV5-10-1	15	Resident
3-2830	1	1	Memory B cell	IGHM	IGHV3-21	21	Resident
3-2839	1	1	Memory B cell	IGHM	IGHV4-34	18	Resident
4-1743	1	1	Memory B cell	IGHM	IGHV3-15	14	Resident
4-3451	1	1	Memory B cell	IGHM	IGHV2-26	13	Resident
4-3470	1	1	Memory B cell	IGHM	IGHV3-49	20	Resident
5-1036	1	1	Memory B cell	IGHM	IGHV5-51	19	Resident
5-4372	1	1	Memory B cell	IGHD	IGHV3-48	20	Resident
5-4790	1	1	Memory B cell	IGHM	IGHV3-53	16	Mobile
5-542	4	1	Memory B cell	IGHM	IGHV3-30	16	Resident
5-796	1	1	Memory B cell	IGHM	IGHV2-5	17	Resident
5-8194	1	1	Memory B cell	IGHM	IGHV3-30	18	Resident
6-1042	1	1	Memory B cell	IGHM	IGHV3-23	16	Resident
6-1379	1	1	Memory B cell	IGHM	IGHV5-51	17	Resident
6-1618	1	1	Memory B cell	IGHM	IGHV3-21	11	Resident
6-1627	1	1	Memory B cell	IGHM	IGHV4-31	15	Resident
6-1755	1	1	Memory B cell	IGHM	IGHV1-69	21	Resident
6-1899	1	1	Memory B cell	IGHM	IGHV1-69	14	Resident
6-2038	1	1	Memory B cell	IGHM	IGHV3-30	16	Resident
6-2517	1	1	Memory B cell	IGHM	IGHV4-31	18	Resident
6-2753	1	1	Memory B cell	IGHM	IGHV3-15	18	Resident
6-3032	1	1	Memory B cell	IGHM	IGHV4-31	22	Resident
6-3069	1	1	Memory B cell	IGHM	IGHV1-2	23	Resident
6-3535	1	1	Memory B cell	IGHM	IGHV4-39	24	Resident
6-3558	1	1	Memory B cell	IGHM	IGHV4-39	23	Resident
6-367	1	1	Memory B cell	IGHM	IGHV4-39	21	Resident
6-4005	1	1	Memory B cell	IGHM	IGHV4-39	13	Resident
6-4218	1	1	Memory B cell	IGHM	IGHV4-34	21	Resident
6-4224	1	1	Memory B cell	IGHM	IGHV1-46	17	Resident
6-4225	1	1	Memory B cell	IGHM	IGHV1-69	12	Resident
6-4233	1	1	Memory B cell	IGHM	IGHV1-18	20	Resident
6-775	1	1	Memory B cell	IGHM	IGHV1-8	21	Resident

Table S8. Table of unmutated IgM/D sequences paired with B cell gene expression data. The clone ID is shown alongside the number of cells belonging to the clone (Cell Count) and the number of V(D)J sequences in the clone that are different (Unique Sequences). Junction length in terms of the number of amino acids in the junction sequence is also shown. Whether the clone can also be traced to the circulation is shown as well (Overlap).

Clone ID	Cell Count	Unique Sequences	Subset	Constant Region	VH Gene	Junction Length	Overlap
3-2833	2	1	Plasma Cell	IGHG1	IGHV4-61	19	Resident
4-3461	1	1	Memory B cell	IGHG1	IGHV1-46	21	Resident
5-5356	1	1	Plasma Cell	IGHG1	IGHV1-3	18	Resident
5-6249	1	1	Plasma Cell	IGHG1	IGHV5-51	17	Mobile
6-1156	1	1	Memory B cell	IGHG3	IGHV3-7	12	Resident
6-1721	1	1	Memory B cell	IGHG1	IGHV4-34	12	Resident
6-2042	1	1	Memory B cell	IGHG3	IGHV1-2	18	Resident
6-2171	1	1	Plasma Cell	IGHG3	IGHV1-69	16	Resident

Table S9. Table of unmutated IgG sequences with paired B cell gene expression data. The clone ID is shown alongside the number of cells belonging to the clone (Cell Count) and the number of V(D)J sequences in the clone that are different (Unique Sequences). Junction length in terms of the number of amino acids in the junction sequence is also shown. Whether the clone can also be traced to the circulation is shown as well (Overlap).

Subject ID	IGH Count	IGH Clones	IgA Sequences	IgG Sequences	IgM Sequences	IgD Sequences	TCRB Count
1	15529	5354	1536	7837	5846	310	27650
2	10827	4194	1932	6105	2566	224	36637
3	8672	2313	1570	5160	1821	121	19795
4	8077	3067	1223	3905	2728	221	15057
5	38035	7535	2070	24795	10927	243	30406
6	11758	3645	605	7842	3043	268	24458

Table S10. Numbers of reads and reconstructed V(D)J sequences from bulk repertoire sequencing of IGH and TCRB sequences from total PBMC RNA derived from Cohort 1 blood samples.

Status	Patient	Largest Clone
EM	1	1
EM	3	2
EM	4	3
EM	5	4
EM	6	184
Uninvolved	1	0
Uninvolved	3	0
Uninvolved	4	0
Uninvolved	5	1
Uninvolved	6	1

Table S11. The size of the largest clone based on the number of cells in that particular clone is shown for uninvolved and EM samples for B cells.

Status	Patient	Largest Clone
EM	1	34
EM	3	10
EM	4	51
EM	5	9
EM	6	12
Uninvolved	1	21
Uninvolved	3	1
Uninvolved	4	2
Uninvolved	5	2
Uninvolved	6	19

Table S12. The size of the largest clone based on the number of cells in that particular clone is shown for uninvolved and EM samples for CD8 T cells.

Status	Patient	Largest Clone
EM	1	12
EM	3	30
EM	4	37
EM	5	9
EM	6	20
Uninvolved	1	5
Uninvolved	3	1
Uninvolved	4	2
Uninvolved	5	3
Uninvolved	6	20

Table S13. The size of the largest clone based on the number of cells in that particular clone is shown for uninvolved and EM samples for CD4 T cells.

Status	Gene	Mean	SD
EM	TLR1	0.452	0.612
EM	TLR2	0.000	0.000
EM	TLR3	0.000	0.000
EM	TLR4	0.000	0.000
EM	TLR5	0.000	0.000
EM	TLR6	0.076	0.092
EM	TLR7	0.081	0.126
EM	TLR8	0.000	0.000
EM	TLR10	0.735	0.307
Uninvolved	TLR1	0.502	0.710
Uninvolved	TLR2	0.000	0.000
Uninvolved	TLR3	0.000	0.000
Uninvolved	TLR4	0.000	0.000
Uninvolved	TLR5	0.000	0.000
Uninvolved	TLR6	0.000	0.000
Uninvolved	TLR7	0.000	0.000
Uninvolved	TLR8	0.000	0.000
Uninvolved	TLR10	0.499	0.706

Table S14. The mean expression (and standard deviation) of TLR family members among B cells from EM or uninvolved skin. Normalized counts of TLR gene transcripts from B cells in a given sample were averaged and the mean ("Mean") and standard deviation ("SD") of these averages was computed for EM or uninvolved skin samples. This is shown as a table. Please note that TLR9 was excluded from analysis owing to its low expression (see Methods, Emulsion-based single-cell library preparation and gene expression analysis).

Status	Gene	Mean	SD
EM	TLR1	0.039	0.023
EM	TLR2	0.227	0.137
EM	TLR3	0.003	0.003
EM	TLR4	0.109	0.051
EM	TLR5	0.015	0.008
EM	TLR6	0.008	0.005
EM	TLR7	0.020	0.012
EM	TLR8	0.035	0.019
EM	TLR10	0.003	0.003
Uninvolved	TLR1	0.056	0.035
Uninvolved	TLR2	0.306	0.097
Uninvolved	TLR3	0.004	0.005
Uninvolved	TLR4	0.092	0.078
Uninvolved	TLR5	0.008	0.011
Uninvolved	TLR6	0.003	0.006
Uninvolved	TLR7	0.003	0.007
Uninvolved	TLR8	0.000	0.000
Uninvolved	TLR10	0.000	0.000

Table S15. The mean expression (and standard deviation) of TLR family members among myeloid cells from EM or uninvolved skin. Normalized counts of TLR gene transcripts from myeloid cells in a given sample were averaged and the mean (“Mean”) and standard deviation (“SD”) of these averages was computed for EM or uninvolved skin samples. This is shown as a table. Please note that TLR9 was excluded from analysis owing to its low expression (see Methods, Emulsion-based single-cell library preparation and gene expression analysis).
